# Breaking the Trade‐Off Between Electrical Conductivity and Mechanical Strength in Bulk Graphite Using Metal–Organic Framework‐Derived Precursors

**DOI:** 10.1002/advs.202416210

**Published:** 2025-01-09

**Authors:** Yuqing Zhang, Junzhuo Wang, Yinghan Zhang, Qi Zheng, Lianjun Wang, Wan Jiang

**Affiliations:** ^1^ State Key Laboratory for Modification of Chemical Fibers and Polymer Materials & College of Materials Science and Engineering Donghua University Shanghai 201620 China; ^2^ Engineering Research Center of Advanced Glasses Manufacturing Technology Ministry of Education Donghua University Shanghai 201620 China; ^3^ Institute of Functional Materials Donghua University Shanghai 201620 China

**Keywords:** electrical conductivity, electromagnetic interference shielding, high‐performance bulk graphite, mechanical strength, metal–organic framework

## Abstract

High‐performance bulk graphite (HPBG) that simultaneously integrates superior electrical conductivity and excellent strength is in high demand, yet it remains critical and challenging. Herein a novel approach is introduced utilizing MOF‐derived nanoporous metal/carbon composites as precursors to circumvent this traditional trade‐off. The resulting bulk graphite, composed of densely packed multilayered graphene sheets functionalized with diverse cobalt forms (nanoparticles, single atoms, and clusters), exhibits unprecedented electrical conductivity in all directions (in‐plane: 7311 S cm⁻¹, out‐of‐plane: 5541 S cm⁻¹) and excellent mechanical strength (flexural: 101.17±5.73 MPa, compressive: 151.56±2.53 MPa). Co nanoparticles act as autocatalysts and binders, promoting strong interlayer adhesion among highly graphitized graphene layers via spark plasma sintering. The strong nano‐interfaces between graphite and Co‐create critical bridges between graphene nanosheets, facilitating highly efficient electron migration and enhanced strength and stiffness of the assembled bulk nanocomposites. Leveraging these exceptional properties, practical demonstrations highlight the immense potential of the robust material for applications demanding superior electromagnetic interference shielding and efficient heating. An innovative approach, which effectively decouples electrical conductivity from mechanical properties, paves the way for the creation of HPBGs tailored for diverse application sectors.

## Graphical abstract

To break the trade‐off between electrical conductivity and mechanical strength of HPBGs, a novel approach utilizing MOF‐derived porous carbon as precursors is developed. The resulting bulk graphite, composed of densely packed multilayered graphene sheets functionalized with diverse cobalt forms, exhibits unprecedented electrical conductivity (7311 S cm^−1^) and excellent mechanical strength, holding immense potential for EMI shielding and Joule heating.

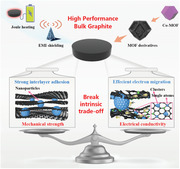

## 1 Introduction

High‐performance bulk graphite (HPBG) has garnered significant attention across diverse sectors, including semiconductors, metallurgy, and aerospace, due to its exceptional properties of low density, high thermal and chemical stability, and excellent electrical conductivity.^[^ ^1^, ^2^, ^3^ ^]^ However, industrial production processes often reduce the electrical conductivity of artificial graphite by 2–3 orders of magnitude compared to pristine graphite. This is due to low graphitization degree, pores, defects, and grain boundaries presented in raw materials like coke and coal tar, which hinder carrier migration.^[^ ^4^, ^5^ ^]^ Moreover, an increase in graphite layers also suppresses interlayer coupling and reduces out‐of‐plane conductivity, limiting HPBG's broader application.^[^ ^6^ ^]^ Another significant obstacle encountered by bulk graphite is its intrinsically low mechanical strength, primarily attributed to the weak van der Waals forces between its layers.^[^ ^7^ ^]^ To address these challenges, researchers have explored strategies involving the incorporation of ultra‐strong components with graphite, as represented by nanodiamonds (NDs). NDs can enhance mechanical properties by establishing covalent bonds between graphite layers and preventing cleavage.^[^ ^8^ ^]^ Additionally, ND‐embedded bulk graphite can be synthesized in situ, leading to a unique composition with both diamond‐like and graphite‐like phases that feature improving overall properties.^[^ ^9^, ^10^, ^11^ ^]^ While NDs enhance mechanical strength, their sp^3^ bonding can limit electrical conductivity (670 to 1240 S m⁻¹).^[^ ^12^ ^]^ This highlights the need to further decouple the mechanical and electrical properties of HPBG materials.

In addition to merging carbon‐based additives with bulk graphite, the incorporation of metals into graphite has emerged as another promising approach to decouple the electrical and mechanical properties.^[^ ^13^, ^14^, ^15^ ^]^ Metals are well known for their ability to catalyze graphitization and increase carrier concentration, leading to substantial improvements in electrical conductivity.^[^ ^16^, ^17^ ^]^ Notably, it has been demonstrated that intercalating nanosized metals (e.g., Ag, Cu) into the multi‐interlayers of graphite can effectively boost the electrical conductivity by over 20 times.^[^ ^18^, ^19^, ^20^ ^]^ Furthermore, metal additives can also mitigate the inherent brittleness of graphite by enhancing its fracture toughness and resistance to crack propagation. The distribution of metal particles within the graphite flake layers effectively reduces interlayer slippage and strengthens the mechanical properties of the bulk material.^[^ ^14^ ^]^ Copper‐based alloys, in particular, have found widespread application in commercial bulk graphite, demonstrating superior electrical conductivity (4000 S cm⁻¹) and flexural strength (117 MPa).^[^ ^21^ ^]^ However, traditional fabrication methods, involving repeated molten metal impregnation into pre‐made graphite matrices, often struggle to control the dispersion of metal particles and the resulting carbon‐metal microstructures.^[^ ^22^ ^]^ This drawback indeed prevents further optimization of electrical conductivity. As metal impregnation is a physical mixing process, we propose that direct transformation of carbon‐based precursors containing well‐dispersed metal sources into HPBG may address the issue of uneven dispersity of metal particles, potentially enhancing electrical conductivity while maintaining mechanical strength.

Metal–organic frameworks (MOFs), archetypal porous crystalline materials constructed from organic ligands and metal ions or clusters through coordination bonds, possess tunable porosity and large surface areas.^[^ ^23^, ^24^, ^25^ ^]^ They have been extensively investigated as precursors for fabricating carbon nanomaterials with well‐defined porous morphologies and monodispersed metallic composites through thermal treatment.^[^ ^26^, ^27^, ^28^ ^]^ These desirable characteristics enable the creation of porous carbon composites with precisely tailored structures and properties.^[^ ^29^, ^30^, ^31^ ^]^ Notably, the atomically distributed metal sites within MOFs facilitate the in‐situ formation of metallic nanoparticles, which exhibit exceptional catalytic activity in transforming amorphous carbon into highly graphitic carbon at low temperatures.^[^ ^32^, ^33^ ^]^ To this end, MOF‐derived metal‐embedded porous carbon is expected to be a promising precursor that can be facilely converted into HPBG materials with well‐decoupled properties.

Another issue associated with traditional metal‐graphite bulk fabrication methods is the high temperature required for both graphite production (>2500 °C) and metal impregnation. Our group has pioneered a well‐established approach for the consolidation of bulk materials under low‐temperature sintering using high‐energy nanostructured porous powders, which are conducive to solid‐state densification.^[^ ^34^, ^35^, ^36^, ^37^, ^38^, ^39^ ^]^ Leveraging this approach, we have successfully fabricated a range of high‐density, functional glass and ceramic materials from molecular sieves, mesoporous silica, and hydroxyapatite precursors under remarkably lower temperatures using spark plasma sintering (SPS).^[^ ^34^, ^37^, ^40^ ^]^ Recently, we extended this strategy to construct high‐strength bulk carbon from ordered mesoporous carbon and ND additives at low temperatures.^[^ ^41^ ^]^ Inspired by these achievements, combining MOF‐derived porous carbon with SPS technology could, in principle, generate HPBG under much milder fabrication conditions.

Herein, we demonstrate the facile fabrication of HPBG materials using MOF‐derived porous carbon nanocomposites as novel precursors, achieving the highest reported electrical conductivity and excellent mechanical properties. A cobalt‐based zeolitic imidazolate framework, ZIF‐67, characterized by its large specific surface area, abundant metal active sites, and facile fabrication, was employed to obtain nanoporous carbon embedded with well‐dispersed cobalt nanoparticles (Co NPs). The resulting high porosity of the porous carbon and the catalytic activity of the Co NPs facilitated rapid self‐densification and graphitization under 1800 °C and 80 MPa within 5 min, a significantly lower temperature than the traditional process for metal‐embedded graphite. The resultant graphite composite bulks are composed of multilayered graphene nanosheets interconnected by Co species, both in‐plane and out‐of‐plane, leading to simultaneous enhancement of electrical conductivity and mechanical strength. Notably, the Co species, primarily Co NPs, along with Co single atoms (SAs) and Co clusters, contribute to the efficient electron migration within the graphite matrix. The bulk graphite composites exhibited extraordinary electrical conductivity in both in‐plane (7311 S cm⁻¹) and out‐of‐plane (5541 S cm⁻¹) directions, surpassing all previously reported graphite bulk composites. Meanwhile, excellent mechanical strength was also achieved, with flexural strength of 101.17±5.73 MPa and compressive strength of 151.56±2.53 MPa. The high performance enables the facile application of HPBG in electromagnetic interference (EMI) shielding and electrothermal conversion. The excellent electrical conductivity endowed the bulk with highly efficient EMI shielding performance of 68.26, 53.52, and 32.45 dB in the X‐band, Ku‐band, and K‐band, respectively. Moreover, an outstanding Joule heating effect was observed, with a surface temperature reaching 80.2 °C under a voltage of only 1.0 V.

## 2 Results and Discussion

### 2.1 Preparation and Structural Characterization

**Figure 1:**
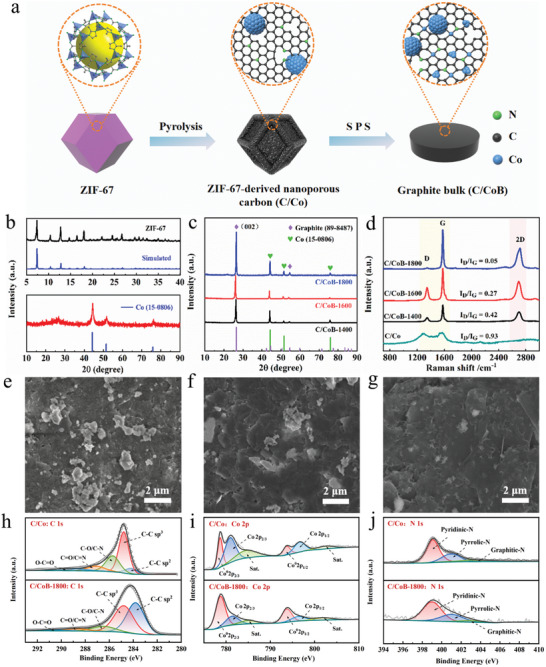
a) Schematic illustration for the fabrication process of C/CoB. b) XRD patterns of ZIF‐67 and C/Co. c) XRD patterns of C/CoB prepared at different sintering temperatures. d) Raman spectra with I_D_/I_G_ values of C/CoB‐1400, C/CoB‐1600, C/CoB‐1800, and C/Co. e–g) Cross‐section SEM images of C/CoB‐1400, C/CoB‐1600, and C/CoB‐1800. h–j) C 1s, Co 2p, and N 1s XPS spectra of C/Co and C/CoB‐1800.

The fabrication process of MOF‐derived Co‐embedded bulk graphite (C/CoB) via SPS is illustrated in **Figure** **1**a. Initially, microcrystalline ZIF‐67 was synthesized through assembly of cobalt ions from cobalt nitrate and organic ligands provided by 2‐methylimidazole, showing typical rhombic dodecahedral morphology in the size of 400–500 nm (Figure S1a, Supporting Information). The corresponding XRD profile matches well with the simulated pattern, indicating phase‐pure MOF synthesis with high crystallinity (Figure 1b).^[^ ^42^ ^]^ Carbonization of ZIF‐67 at 700 °C in an inert atmosphere yielded Co embedded porous carbon (C/Co) via a morphology‐preserved thermal transformation process, in which Co ions were reduced to metallic nanoparticles (NPs) and organic ligands turned to nitrogen‐doped amorphous carbon framework (Figures S1b and S2, Supporting Information). The XRD pattern of C/Co shows three diffraction peaks at 44.2°, 51.6°, and 76.0°, corresponding to the (111), (200), and (220) planes of crystalline cobalt with face‐centered cubic (FCC) structure (Figure 1b).^[^ ^43^ ^]^ Moreover, the hexagonal close‐packed (HCP) phase of cobalt is also identified (Figure S4, Supporting Information). The porous nature of C/Co was analyzed by N_2_ adsorption‐desorption isotherms (Figure S3, Supporting Information). The isotherm manifests an H4‐type hysteresis loop, suggesting a typical mesoporous structure with a Brunauer‐Emmett‐Teller (BET) surface area of 176.7 cm^2^ g^−1^.^[^ ^44^ ^]^ The average pore size distribution of 3.8 nm further confirmed the formation of mesopores. The ingenious arrangement of metallic NPs within a well‐organized porous carbon framework renders itself a high‐energy precursor for the formation of densified graphite composite bulks via phase transition induced by elevated temperature and pressure. The C/Co powder was subjected to a SPS process with a pressure of 80 MPa and temperature of 1400, 1600, and 1800 °C, respectively, for 5 mins. As shown in XRD patterns, the (002) diffraction peak corresponding to graphite at 26.5° indicated amorphous carbon was successfully converted to graphite (Figure 1c). The samples obtained from 1400 °C, 1600 °C, and 1800 °C are denoted as C/CoB‐1400, C/CoB‐1600, and C/CoB‐1800, respectively. With the increased sintering temperature, the diffraction peak at 26.5° enhanced, suggesting an increased degree of crystallization of graphite. The degree of graphitization at different sintering temperatures was calculated to be 93%, 94%, and 97%, respectively. These results demonstrate that using ZIF‐67‐derived Co/C as a raw material triggers the production of bulk graphite with a high graphitization degree without undergoing long‐term high‐temperature treatment.

Figure 1d displays the Raman spectra for the C/Co and C/CoB. Three distinct peaks at ≈1350 cm^−1^ (D band), ≈1580 cm^−1^ (G band) and ≈2714 cm^−1^ (2D band) were observed.^[^ ^45^ ^]^ The D band reveals the local defects and the disordered carbon, while the G band corresponds to the planar vibrations of graphite with lattice structure and the 2D band refers to the characteristic peak of graphene.^[^ ^46^ ^]^ The Raman spectra further confirm the transformation of amorphous carbon of C/Co powder to graphite in C/CoB after SPS treatment. Meanwhile, with the increase of sintering temperature, the D band decreases while the G band and 2D band dramatically rise. The intensity ratio of D band and G band (I_D_/I_G_) shows a progressive decrease from 0.42 to 0.05 ranging from C/Co‐1400, C/Co‐1600 to C/Co‐1800, indicating high temperature induced the formation of oriented and highly graphitized crystallites.^[^ ^47^ ^]^ The presence of a strong 2D band in C/CoB samples suggests the Co‐embedded bulk graphite is assembled by multilayer graphene, which is beneficial for the enhancement of electrical conductivity. The blue shift of C/CoB‐1800 compared with the other two samples further confirms the improved graphitization degree, which is in agreement with XRD results. The fracture surfaces of C/CoB were revealed by SEM images in Figure 1e,g. The cross‐section of C/Co‐1400 presented a rough surface with unevenly distributed Co particles (Figure 1e). With the increase of sintering temperature, the number of metallic fragments reduced, and the fracture surface turned smoother (Figure 1f). Upon heating to 1800 °C, the surface exhibited much‐improved homogeneity and uniform density (Figure 1g). This can be ascribed to the high sintering temperature of 1800 °C that exceeds the melting point of cobalt (1495 °C) leads to the diffusion of Co within the carbon matrix along with the elevated pressure. The melted Co can be regarded as binders that connect graphite flakes to form highly compact bulks, which is beneficial for improved electronic conductivity as well as mechanical strength typically along dense planes.

X‐ray photoelectron spectroscopy (XPS) analysis was performed to further investigate the effect of SPS on the chemical states of the component. The survey spectrum confirms the existence of C, N, Co, and O in the C/Co and C/CoB (Figure S5, Supporting Information). As presented in Figure 1h, the five deconvoluted peaks of C 1 s located at 284.1, 284.8, 285.7, 287.1, and 289.3 eV, which link to sp^2^‐hybridized C, sp^3^‐hybridized C, C─N (or C─O) bond, C═N (or C═O) and O─C═O bond, respectively.^[^ ^48^, ^49^ ^]^ It is clear to see that compared with C/Co, the XPS area of sp^3^‐hybridized carbon atoms in C/CoB decreased accompanied by the increase of sp^2^‐hybridized carbon atom, indicating the more complete sp^2^‐conjugated structure is formed in the bulk composites, which is consistent with XRD and Raman analysis. Besides, the XPS peaks of N1s (Figure 1j) at 399.0, 400.9, and 403.4 eV in the samples are assigned to the pyridinic‐N, pyrrolic‐N, and graphitic‐N, respectively.^[^ ^50^ ^]^ The presence of C─N and C═N bonds indicate that nitrogen was doped into carbon structures and pyrrolic‐N is the dominant species in the bulk samples. Meanwhile, the chemical states of Co species were also verified. For Co/C, the peaks located at ≈778.5 eV (Co 2p_3/2_) and ≈793.5 eV (Co 2p_1/2_) in the high‐resolution Co 2p spectrum indicate the presence of metallic cobalt, and those peaks located at ≈781.0 and ≈796.5 eV originated from Co 2p_3/2_ and Co 2p_1/2_, respectively, suggesting the existence of Co^2+^ (Figure 1i).^[^ ^51^, ^52^ ^]^ In contrast, for C/CoB‐1800, the high‐resolution Co 2p spectrum clearly shows the Co 2p_3/2_ and Co 2p_1/2_ peaks centered at 778.8 and 793.8 eV, respectively, are lower than those of reported Co 2p_3/2_ (780.9 eV) and Co 2p_1/2_ (796.2 eV) for Co^2+^ and higher than those of Co 2p_3/2_ (778.5 eV) and Co 2p_1/2_ (793.5 eV) for Co^0^, suggesting the chemical states of Co in C/CoB‐1800 is between 0 and +2.^[^ ^53^ ^]^ Considering the nitrogen‐doped carbon support derived from the organic ligands in ZIF‐67 suggested in the XPS results, we suspect some Co species are presented as single atoms anchored in the N‐doped carbon matrix.

**Figure 2:**
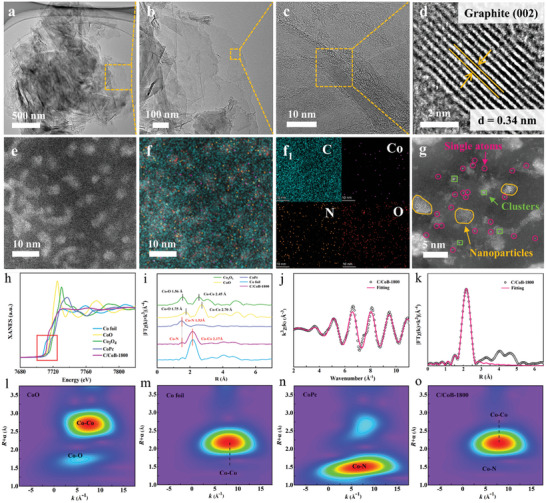
Microstructural characterizations and chemical structure of C/CoB‐1800. a) TEM image. b) Detailed magnified TEM image. c) HRTEM image. d) Detailed magnified HRTEM image. e) HAADF‐STEM image of carbon matrix regions. f‐f_1_) Elemental mapping images of Co, N, C, and O. g) Detailed magnified HAADF‐STEM image of Co. h) Co *K*‐edge XANES spectra, i) EXAFS spectra in R space of Co foil, CoPc, Co_3_O_4_, CoO, and C/CoB‐1800. j) Co k^3^ χ(k) oscillation curves, k) Fourier transform of the EXAFS spectra and the corresponding fitting curve of C/CoB‐1800. l–o) Wavelet transforms (WT) of CoO, Co foil, CoPc, and C/CoB‐1800.

The microstructure of C/CoB‐1800 was further analyzed by transmission electron microscopy (TEM) and high‐resolution high‐angle annular dark field scanning transmission electron microscopy (HAADF‐STEM) images. As shown in **Figure** **2**a–c), the prepared graphite composite bulks are assembled by multilayered graphene nanosheets with the edge comprising ≈10–15 layers. The crystal plane spacing of 0.34 nm is indexed to the (002) plane of graphite, further convincingly confirming the amorphous carbon derived from ZIF‐67 is converted to graphite (Figure 2d). HAADF‐STEM images of C/CoB‐1800 were carried out to have a deep understanding of the distribution of Co species at the atomic scale (Figure 2e). To eliminate grain overlaps in STEM imaging, lamellae with a thickness of ≈50 nm were cut with a focused ion beam (FIB) (Figure S6, Supporting Information). When checking the sintered bulk with aberration‐corrected scanning transmission electron microscopy, interestingly, in addition to Co nanoparticles (NPs), Co single atoms (SAs) and Co clusters are distributed in a graphite matrix, which is labeled with pink circles and green boxes (Figure 2g; Figure S7, Supporting Information). Figure 2(f‐f_1_) shows the representative Co, N, and C mappings by electron energy‐loss spectroscopy (EELS). Distribution of Co was observed in the carbon matrix, ascribed to the presence of Co SAs and Co clusters. Moreover, N heteroatoms are homogeneously distributed over the entire architecture, originating from the decomposition of N‐containing linkers.^[^ ^54^ ^]^ To further verify the presence of Co SAs and clusters in the bulk graphite, we performed X‐ray absorption near‐edge spectroscopy (XANES) and extended X‐ray absorption fine structure (EXAFS) to investigate the local electronic structures and geometric structures of the central Co atoms in C/CoB‐1800. As seen in Figure 2h, the Co K‐edge absorption edge of C/CoB‐1800 is located between those of CoO and Co foil, further revealing the average chemical valence state of Co is between 0 and +2.^[^ ^55^ ^]^ An extended EXAFS Fourier transform (FT) was used to characterize the local coordination environment of Co within C/CoB‐1800. As illustrated in Figure 2i, a predominant peak at 2.17 Å corresponds to Co─Co bonds, confirming the existence of Co NPs and Co clusters. The weak Co─N coordination at ≈1.53 Å implied the existence of Co SAs in C/CoB‐1800. To identify the coordination configuration of the central Co atoms, the EXAFS spectra were fitted. Based on the fitting results of Co foil, Co_3_O_4_, CoPc, CoO, and C/CoB‐1800 as well as their scattering paths, it can be inferred that the coordination number of Co species in C/CoB‐1800 is ≈3 and the Co‐Co bond length is ≈2.48 Å (Figure 2j,k; Figure S8 and Table S1, Supporting Information). A lower coordination number of Co‐N bonds located at 1.90 Å demonstrates Co atoms are anchored to the N‐site as single atom form.^[^ ^56^ ^]^ These structural features together validate the existence of the Co SAs, clusters, and NPs in C/CoB‐1800. In addition, the wavelet transform (WT) of Co *K*‐edge EXAFS oscillation was applied to further analyze the atomic information. The WT of the C/CoB‐1800 contour plot exhibits an intensity maximum at ≈7.80 Å^−1^ in k space that is assigned to Co─Co, and a second intensity maximum at ≈5.60 Å^−1^ originating from Co─N scattering (Figure 2o). The signal corresponding to the Co─N bond is observed to be overlapped by that of the Co─Co bond due to the lesser amount of Co SAs than that of Co clusters and Co NPs. These results confirm the co‐existence of Co─Co and Co─N bonds in C/CoB‐1800, which further approves Co SAs, Co clusters, and Co NPs in the highly dense graphite composite bulks via high temperature and pressure. The transformation of Co NPs to clusters and SAs is quite interesting, as in general, metallic NPs tended to aggregate under elevated temperatures and pressures, driven by the decrease of metal surface free energy. The plausible conversion mechanism is proposed as follows. During the annealing treatment under the N_2_ environment, the organic linkers of ZIF‐67 undergo a pyrolysis process to generate N‐doped porous carbon, and the cobalt nodes are reduced by the generated carbon.^[^ ^57^ ^]^ The further elevated temperature and pressure during the SPS process lead to the diffusion of Co NPs and partial emission of Co atoms. The resulting mobile Co atoms were subsequently captured by N defects on carbon support driven by the thermodynamically stable coordination between Co and N.^[^ ^58^ ^]^

### 2.2 Mechanical, Electrical, and Thermal Properties

**Figure 3:**
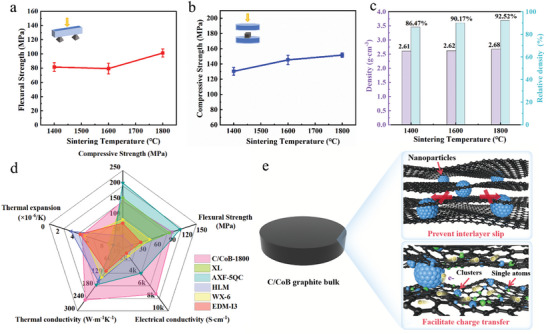
a) Flexural strength, b) Compressive strength of C/CoB‐1400, C/CoB‐1600 and C/CoB‐1800. c) Density and relative densities of C/CoB at different sintering temperatures. d) Radar map comparison of physical properties between commercial HPBGs and C/CoB‐1800 (Table S4, Supporting Information). e) Schematic illustration of the microstructure of C/CoB.

The mechanical properties of the prepared C/CoB bulks are greatly strengthened by the firm anchor of Co species between layered graphene microstructure in the graphite matrix. It is apparent the mechanical performance of the prepared nanocomposite bulks is positively correlated with the sintering temperature. Specifically, the C/CoB fabricated at 1800 °C achieved an optimal flexural strength of 101.17±5.73 MPa, compressive strength of 151.56±2.53 MPa, and Young's modulus of 12.72±1.84 GPa (**Figure** **3**a,b; Figure S10, Supporting Information). The corresponding stress‐strain curves are characterized in Figure S11 (Supporting Information). The flexural and compressive strength of the C/CoB‐1800 are significantly higher than those of commercially available graphite materials.^[^ ^59^, ^60^ ^]^ The markedly improved strength is closely related to the densely packed graphene sheets “glued” by Co moieties. As shown in Figure 3c, the relative density of C/CoB increased with the rising sintering temperature and achieved 92.52% at 1800 °C. The densely layered bulk structure is clearly indicated by the material cross‐section images shown in Figure 1g. The shrinkage curve serves as a good tool to monitor the densification of the material during the sintering process. The variations of shrinkage displacement and temperature with time during the in situ reaction sintering of C/Co powders are shown in Figure S12 (Supporting Information). Notably, the C/Co powders undergo significant volumetric shrinkage when the pressure increases from 50 to 80 MPa, corresponding to the temperature of 1200–1300 °C. The maximum relative shrinkage rate reaches ≈1.5%, indicating an increased stacking density. Given the rapid heating rate of 100 °C min^−1^, this shrinkage is completed within just one minute. MOF‐derived porous carbon skeleton rapidly collapses and undergoes a local rearrangement of carbon atoms from sp^3^ to sp^2^ bonding catalyzed by Co NPs during this stage. Moreover, under high pressure, Co NPs break into smaller grains and fill voids between assembled graphitic layers. Subsequently, as the temperature continues rising, the consolidation of the C/Co continues but the relative shrinkage rate becomes stabilized. When the sintering temperature reaches 1800 °C, the bulk completes the densification and ultimately maximizes the mechanical properties. For graphite materials with a layered structure, the cracks usually extend along the slip plane. Nevertheless, in the case of C/CoB‐1800 nanocomposites, the metallic NPs or clusters embedded between the graphene layers effectively prevented further crack propagation, altering the interlayer fracture mode and remarkably improving mechanical properties (Figure 3e).^[^ ^61^ ^]^ The hybridization of sp^3^ and sp^2^ carbon atoms additionally contributes to the high mechanical strength and modulus of the bulk.^[^ ^12^ ^]^

The electrical conductivity of C/CoB was evaluated using a standard four‐probe method. The results demonstrated that the increasing temperature enhances electron conduction in both the in‐plane and out‐of‐plane directions of the prepared bulk graphites (Table S3, Supporting Information), consistent with the graphitization degrees determined by Raman spectroscopy and XRD. Notably, C/CoB‐1800 exhibited exceptional room‐temperature electrical conductivity of 7311 S cm⁻¹ (in‐plane) and 5541 S cm⁻¹ (out‐of‐plane), significantly exceeding the commercially available graphite (Table S4, Supporting Information). The conductive pathway constructed by the highly graphitized (≈98%) graphene layers facilitated electron migration throughout the basal plane (Figure 3e). Furthermore, the in situ formed well‐dispersed metallic Co species create a micro‐nano synergistic conductive network in conjunction with stacked graphene sheets, which additionally fill the network between graphene nanosheets with the aid of high pressure, leading to efficient carrier mobility along prismatic planes (Figure 3e). The critical role of MOF precursors in achieving ultra‐high conductivity of the graphite bulk composite was also investigated. Commercial graphite powder and porous carbon nano‐powder were physically mixed with nanosized metallic cobalt, respectively, and the corresponding composite bulks sintered at the same condition with C/CoB‐1800 were named GM‐1 and GM‐2, respectively. In contrast with C/CoB‐1800, Co NPs in GM‐1 and GM‐2 were observed to be severely agglomerated and graphite flakes were in loose contact with each other (Figure S14, Supporting Information). The poorly ordered microstructure of GM‐1 and GM‐2 resulted in low electrical conduction capability, in which GM‐2 only exhibited 151 S cm⁻¹ along the in‐plane direction (Table S5, Supporting Information). These results provide direct evidence for the positive effect of MOF‐derived metal‐embedded porous carbon on producing graphite nanocomposite bulks with outstanding electrical conductivity both in‐plane and out‐of‐plane direction.

The highly organized and highly crystallized graphitic structures also endow C/CoB‐1800 with excellent thermal conductivity. The out‐of‐plane thermal conductivity of C/CoB‐1800 reaches 91 Wm^−1^K^−1^, as temperature increases, the out‐of‐plane thermal conductivity decreases, while the in‐plane thermal conductivity reaches a maximum value of 248 Wm⁻¹K⁻¹ at 423 K (Figure S15, Supporting Information). Additionally, the coefficient of thermal expansion (CTE) is crucial for preventing thermal cracks at the interface of heterogeneous composites. The out‐of‐plane CTE of C/CoB‐1800 achieves 4.50 × 10^−6^ K^−1^. Based on the results, C/CoB‐1800 demonstrates overall outstanding performance regarding electrical, thermal conduction as well as mechanical strength compared with commercially available bulk graphite (Figure 3d; Table S4, Supporting Information), rendering their potential applications in electronic devices, thermal management, etc.

### 2.3 EMI Shielding Performance

**Figure 4:**
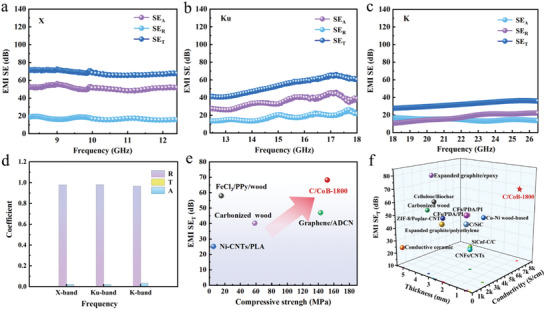
EMI shielding performance of C/CoB‐1800 in the a) X‐band, b) Ku‐band, and c) K‐band. d) The R, T, and A coefficients of C/CoB‐1800. e) The compressive strength and EMI SE_T_ value of C/CoB‐1800 compared with reported carbon‐based EMI shielding bulks (Table S9, Supporting Information). f) EMI shielding performance and electrical conductivity of typical bulk materials compared with C/CoB‐1800 (Table S7, Supporting Information).

Utilizing the superior electrical conductivity of C/CoB‐1800, we investigated its potential application in EMI shielding. **Figure** **4**a characterizes the EMI SE in the frequency range of 8.2‐12.4 GHz (X‐band). The total shielding effectiveness (SE_T_) reaches 68.26 dB, and the reflection efficiency (SE_R_) and absorption efficiency (SE_A_) values, derived using the measured s parameters, are 51.35 and 16.91 dB, respectively. Similarly, C/CoB‐1800 exhibits excellent overall EMI shielding performance in 12.4–18 GHz (Ku‐band) and 18–26.5 GHz (K‐band), respectively, with EMI SE_T_ values reaching 53.32 dB and 32.45 dB (Figure 4(b,c) and Table S6, Supporting Information), well exceeding the EMI SE (20 dB) required in commercial applications.^[^ ^62^ ^]^ These characteristics enable C/CoB‐1800 as a robust and ultra‐broadband EMI shielding material covering 8.2–26.5 GHz. To further analyze the shielding mechanism, the average values of reflection coefficient (R), absorption coefficient (A), and transmission coefficient (T) of C/CoB‐1800 were calculated. As illustrated in Figure 4d, the R values constantly exceed the A value in the whole range of 8.2‐26.5 GHz, indicating that reflection is the primary shielding mechanism due to the impedance mismatch between free space and the bulk surface.^[^ ^63^ ^]^ The thickness of the C/CoB is also an important factor affecting the electromagnetic shielding effect. In this work, the SE/t values of X, Ku, and K‐band are 92.20, 72.32, and 43.85 dB mm^−1^, respectively. Furthermore, the specific EMI SE (SSE/t = SE/density/thickness) values are also critical indicators to evaluate EMI shielding performance. The C/CoB‐1800 boasts an impressive SSE/t value of 344.19 dB cm^2^ g^−1^ in the X‐band, 268.86 dB cm^2^ g^−1^ in the Ku‐band, and 163.62 cm^2^ g^−1^ in the K‐band, respectively. Compared to recently reported carbon‐based materials, C/CoB‐1800 bulk demonstrates exceptionally efficient EMI shielding performance across the full 8.2–26.5 GHz at a very thin thickness (Table S7 and Table S8, Supporting Information). Additionally, it also exhibits an excellent integration of EMI SE_T_ and mechanical properties compared with rigid carbon‐based bulk materials (Figure 4e; Table S9, Supporting Information). The integration of multiple remarkable features renders C/CoB‐1800 a promising candidate for applications requiring robust EMI protection, including aerospace, military, civil buildings, etc (Figure 4f).

The excellent EMI shielding characteristic is directly related to the electrical conductivity and structural composition of C/CoB‐1800 bulk. When the EMW is incident to the surface of the bulk, the abundant free electrons associated with the ultra‐high conductivity of the composite result in the reflection of most of the EMWs. The in situ‐formed highly graphitic network assembled by multilayered graphene sheets forms a continuously conductive network, which facilitates electron migration and generates conduction loss that converts electromagnetic energy into heat energy.^[^ ^64^ ^]^ Besides, the regular graphite layered structure facilitates multiple reflections of EMWs within the composites. The magnetic metal derived from ZIF‐67 provides magnetic loss (Figure S16, Supporting Information), further enhancing EMWs attenuation within the material. Moreover, the various forms of metal within the carbon matrix contribute to a variety of polarization relaxation processes. The Co SAs and Co clusters distributed in the carbon matrix serve as polarization centers, resulting in the imbalance of dipole moment and the formation of dipole polarization.^[^ ^65^ ^]^ Heterogeneous interfaces between Co NPs and graphitic carbon produce space charges that gather at these interfaces, generating interfacial polarization loss, which further promotes the dissipation of EMWs.^[^ ^66^ ^]^ Furthermore, the highly dense structure of the C/CoB‐1800 composite can additionally enhance the reflection and scattering of EMWs, thereby effectively weakening the propagation ability of the incident microwaves. As such, the highly conductive network together with abundant interfaces and dielectric/magnetic species within the graphite matrix ultimately leads to the excellent EMI shielding properties of C/CoB‐1800 in a wide range of frequencies.

### 2.4 Joule Heating Performance

**Figure 5:**
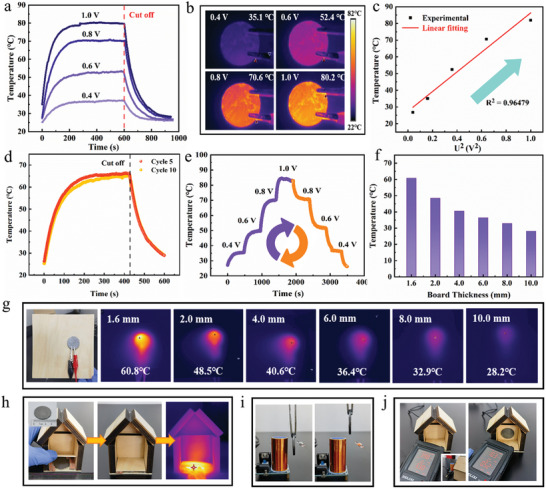
a) The temperature‐time curves of C/CoB‐1800 at different input voltages. b) Infrared images of C/CoB‐1800 at various input voltages. c) Experimental data and linear fitting of surface temperature to U^2^. d) Cyclic stability of C/CoB‐1800 at cycles 5 and 10 at 0.8 V. e) Surface temperature of C/CoB‐1800 with gradient voltage variation. f) Distribution diagram of the relationship between wood thickness and surface temperature at 0.8 V energized for 5min. g) Infrared images of the surface temperature of the back of the wooden board with various thicknesses. h) Schematic diagram of a bulk used for modeling wood floor heaters in houses. i) Images depicting changes in an incandescent bulb without or with the C/CoB‐1800 under the influence of a Tesla coil. j) Electric and magnetic field values of the house before and after the installation of the C/CoB‐1800.

Considering the ultrahigh electrical conductivity and excellent thermal conductivity, the C/CoB‐1800 bulk was expected to exhibit remarkable joule heating properties. The temperature variation of the C/CoB‐1800 was measured by applying different fixed voltages. **Figure** **5**a illustrates the temperature change curves of the C/CoB‐1800 surface under various voltages. It can be observed that the surface temperature of the C/CoB‐1800 increases with the input voltage, and remarkably, reaches the equilibrium temperature of 35.1, 52.4, 70.6, and 80.2 °C at low voltages of 0.4, 0.6, 0.8, and 1.0 V, respectively. The surface temperature of bulk graphite at an applied voltage of only 1.0 V exceeds that of typical carbon‐based materials reported in recent years (Table S10, Supporting Information), demonstrating its superior electrothermal properties. Moreover, when the direct current voltage was turned off, the bulk exhibited fast cooling behavior, demonstrating efficient and reversible electrothermal conversion performance (Figure 5b). The heating temperature of the C/CoB‐1800 had a linear relationship with the square of the voltage (U^2^), as shown in Figure 5c, indicating good heating controllability could be achieved by adjusting the applied voltage.^[^ ^44^ ^]^ The excellent durability and stability of C/CoB‐1800 are further shown by electrothermal cycling stability tests (Figure 5d). In addition, as depicted in Figure 5e and C/CoB‐1800 possesses high sensitivity to input voltages and exhibits excellent electrothermal conversion characteristics. These data indicate the prepared C/CoB‐1800 is of high efficiency and cyclic stability in electrothermal performance, together with its excellent thermal conductivity, endowing C/CoB‐1800 with tremendous potential in practical daily applications, for instance, underfloor heating. Figure 5g,f shows the surface temperature of the back of the wooden boards with different thicknesses heated by C/CoB‐1800 under 0.8 V for 5 mins. For the board with a thickness of 1.60 mm, the resulting temperature can reach 60.80 °C. It is apparent that with the increase in the board thickness, the surface temperature decreased. However, even for boards with thicknesses of 8 and 10 mm, which are commonly used as floors for housing, the surface temperature can still reach a comfortable temperature for humans quickly, demonstrating its potential in practical energy‐efficient heating.

In order to evaluate the joule heating and EMI shielding performance of C/CoB‐1800, a model simulating the real‐world scenario was constructed (Figure 5h). The bulk was placed under a wooden floor with a commercially standard thickness of 8 mm and subjected to an applied voltage of 0.8 V, the indoor floor temperature increased significantly above the ambient temperature creating a noticeable temperature difference. In addition, a wireless power transfer system using a BD243 Tesla coil (Figure S17, Supporting Information) with an operating frequency of approximately 6.36 MHz was employed as the EM emission source.^[^ ^67^ ^]^ When the Tesla coil was activated, an LED bulb lit up. However, when the C/CoB‐1800 was positioned between the Tesla coil and the LED bulb, the bulb was extinguished instantly (Figure 5i). This observation demonstrates that the C/CoB‐1800 bulk effectively shields EMW signals, thus successfully realizing EMI shielding. Furthermore, to simulate real‐world scenarios, when the Tesla coil was activated, the EM radiation inside the house reached values of 398 V m^−1^ and 3.30 µT (Figure 5j). Subsequently, placing the C/CoB‐1800 under the wooden board rapidly reduced the magnetic and electric field values inside the house to 103 V m^−1^ and 0.52 µT, respectively, as shown by the EM radiation meter. These findings confirm the highly conductive C/CoB‐1800 can serve as multifunctional bulks, exhibiting excellent EMI shielding and efficient electrothermal performance for human EM radiation protection and indoor heating, etc.

## 3 Conclusion

This study demonstrates the potential of MOF‐derived porous carbon composites as high‐energy precursors for the facile fabrication of graphite composite bulks coupling exceptional electrical conductivity and strong mechanical strength. As a proof‐of‐concept, nanoporous carbon embedded with Co NPs, derived from ZIF‐67 – a prototypical cobalt‐based MOF – was utilized. During sintering, the porous carbon skeleton undergoes rapid collapse and in situ assembly into densely packed, multilayered graphene sheets, catalyzed by the Co NPs. The dispersed Co NPs provided strong interlayer adhesion and interaction, enhancing both in‐plane and out‐of‐plane conductivity and preventing cleavage along the dense planes. Intriguingly, Co NPs were observed to partially diffuse as single atoms and clusters within the graphite lattice phase, further facilitating efficient electron migration. Consequently, the resultant graphite composite bulks demonstrate remarkable electrical conductivity, with in‐plane and out‐of‐plane values reaching 7311 and 5541 S cm^−1^, respectively. Sufficient mechanical strength with flexural and compressive strengths of 101.17±5.73 MPa and 151.56±2.53 MPa, respectively were achieved. The highly organized graphitic structures further impart the graphite composite bulk with superior thermal conductivity, with an in‐plane thermal conductivity peaking at 248 Wm⁻¹K⁻¹ at 423 K. Given their exceptional overall properties, practical application demonstrations were conducted to highlight the immense potential of these robust materials for applications requiring superior EMI shielding and efficient heating. The graphite composite bulk exhibited highly efficient EMI shielding performance, with values of 68.26, 53.52, and 32.45 dB in the X‐, Ku‐, and K‐band, respectively. Additionally, the bulk composite demonstrated an excellent Joule heating effect, achieving a surface temperature of 80.2 °C under a voltage of only 1.0V. We anticipate that the material developed in this study, along with the concept of utilizing MOF‐derived porous carbon as innovative raw materials for high‐performance bulk graphite, represents a significant advancement in the design of strong and conductive materials for diverse applications in nanomechanics, aerospace, electronics, and beyond.

## 4 Experimental Section

### Materials

Cobalt nitrate hexahydrate (Co(NO_3_)_2_^.^6H_2_O, AR), 2‐Methylimidazole (AR), and Methanol (AR) were purchased from Aladdin (Shanghai, China). All chemicals were analytical grade and were used directly without further purification. Commercial graphite powder, metallic cobalt powder, and nanocarbon powder used for control experiments were purchased from Shanghai Macklin Biochemical Co., Ltd. The purity of cobalt powder is 99.9% with a particle size of 280 mesh. The graphite powder, with a purity of 99.5%, exhibited a specific surface area of 14.9 cm^2^ g^−1^ and an average pore diameter of 3.8 nm. The nano carbon powder (99.5% purity) exhibited an average particle size of 30 nm, a pore diameter of 46.1 nm, and a specific surface area of 114.8 cm^2^ g^−1^.

### Preparation of C/Co

Co(NO_3_)_2_^.^6H_2_O (8.148 g) was dissolved into 350 mL of methanol. Separately, 2‐Methylimidazole (9.1952g) was dissolved into 350 mL of methanol and subsequently poured into the pink cobalt nitrate solution. The mixture solution was stirred for 24 h at room temperature. The purple solids were collected by centrifuging and washed with methanol three times and dried at 80 °C in air for 6 h. The ZIF‐67 precursors were pyrolyzed at 700 °C (5 °C min^−1^) for 2 h under an argon atmosphere. Finally, ZIF‐67‐derived porous carbon (C/Co) was obtained.

### Preparation of C/CoB

The powders obtained after pyrolysis (C/Co) were loaded into graphite dies with inner diameters of 10, 15, and 30 mm, and then sintered using a spark plasma sintering apparatus (Fuji Electronic Industrial Co., Ltd., SPS‐725, Japan) at temperatures ranging from 1400 to 1800 °C under a pressure of 80 MPa. Sintering was performed in a vacuum atmosphere with a heating rate of 100 °C min^−1^, starting from 507 °C. The material was held at the target temperature and pressure for 5 min, then cooled to room temperature. The obtained bulks were denoted as C/CoB‐1400, C/CoB‐1600 and C/CoB‐1800.

### Preparation of GM‐1 and GM‐2

Commercial graphite powder and carbon powder were mixed with metallic cobalt powder according to the mass fractions of Co and C in C/CoB‐1800. The mixtures were then sintered at 1800 °C under a pressure of 80 MPa. The resulting bulks were designated as GM‐1 and GM‐2, respectively.

### Characterization

The structure was investigated by X‐ray diffraction (XRD) using a DX‐2700B X‐ray diffractometer with a Cu Kα radiation source (40 kV, 200 mA). The morphology and microstructures of the above samples were characterized using scanning electron microscopy (SEM, TESCAN VEGA) and transmission electron microscopy (TEM, FEI Talos F200SS). A spherical aberration‐corrected transmission electron microscope (STEM, Thermo Scientific, Themis Z, USA) was used to observe the forms of metal Co. To eliminate grain overlaps in STEM imaging, the C/CoB‐1800 bulk was cut into foils with a thickness of ≈50 nm using a focused ion beam scanning electron microscope (FIB‐SEM, Thermo Helios 5 CX, USA). X‐ray photoelectron spectroscopy (XPS) was measured by ESCALab MKII X‐ray photoelectron spectrometer using Al Ka X‐ray as the excitation source. Raman spectra were characterized on a Raman spectrometer (inVia Reflex, λ = 532 nm). The N_2_ adsorption‐desorption curves were examined by a Quadrachrome adsorption instrument (Quantachrome Autosorb‐iQ, USA) and the specific surface area was evaluated by the Brunauer‐Emmett‐Teller (BET) method. The quantitative analysis of elements was determined by an inductively coupled plasma optical emission spectrometer (ICP‐OES, Leeman Prodigy, USA) analysis system. The X‐ray absorption spectroscopy (XAS) experiments were carried out at the 02B02 beamline of the Shanghai Synchrotron Radiation Facility (SSRF). Co‐K‐edge XANES data was recorded in transmittance mode. The energy calibration of the sample was conducted through standard and Co foil, which as a reference was simultaneously measured. The x‐ray absorption spectra were obtained using the BL01C1 beamline at NSRRC, where the radiation was monochromatized with a Si (111) double‐crystal monochromator. Athena and Artemis software were used to simplify and analyze the XANES and EXAFS data. The magnetic performance was characterized by a vibrating sample magnetometer (VSM) system (manufactured by Lakeshore, Inc.). The surface temperature of the samples was measured by an infrared thermal imager for 346L‐GT from FOTRIC INC.

### Mechanical, Electrical, and Thermal Conductivity Measurement

The compressive strength and flexural strength of C/CoB were evaluated using an electric universal testing machine (UTM; Shimadzu, AGS‐10KNG, Japan). In the compression experiments, the samples were machined to 2 mm × 2 mm × 2 mm, and the flexural strength of C/CoB (12 mm × 2 mm × 2 mm) was measured by a three‐point bending test. Each test was repeated three times, and the average values were reported along with the standard deviations. The electrical conductivity was measured using a four‐point probe tester, the Equation (1) is shown as follows:

(1) $$\sigma =\frac{L}{S\times R}\;$$

where R denotes the resistance of the sample, L and S represent the length and cross‐sectional area of the sample, respectively.

The thermal conductivity was obtained using the following Equation (2):

(2) $$T_{C}=\alpha C_{P}\rho$$

where α denotes the thermal diffusivity, which was measured by a laser thermal conductivity instrument (LFA457). The specific heat capacity (*C*_p_) of C/CoB‐1800 was tested using a differential scanning calorimeter (DSC204F1) from 20 to 500 °C with a heating rate of 10 min^−1^ under a nitrogen atmosphere. The densities of the samples were measured by $\rho =\frac{m}{V}$. The linear coefficient of thermal expansion (CTE) of the C/CoB‐1800 (15mm × 3 mm × 3 mm) was tested using a thermal mechanical analyzer (DIL 402C).

The degree of graphitization was calculated using the model proposed by Maire and Mering. The Equation (3) is shown as follows:

(3) $$g\;\left(\%\right)=\frac{0.3440-d_{\left(002\right)}}{0.3440-0.3354}\;\times 100$$

where g is the degree of graphitization, *d*_(002)_ is the interlayer spacing derived from the Bragg equation and the value of *d*_(002)_ was accurately obtained using silicon calibration of XRD.

(4) $$d_{\left(002\right)}=\lambda /2sin\theta_{002}$$

### EMI Shielding Measurements

The EMI shielding performance of the samples was characterized using a vector network analyzer (N5234B, USA), which measured the scattering parameters (S11 and S21). The C/CoB‐1800 bulks (22.9 mm × 10.2 mm, 15.9 mm × 8.03 mm, and 10.95 mm × 4.5 mm) were examined in the X‐band (8.2‐12.4 GHz), Ku‐band (12.4–18 GHz), and K‐band (18.0–26.5 GHz), respectively, using the waveguide method. Transmission line theory is used to explain the EMI shielding mechanism. When electromagnetic waves are radiated to the shielding material, there are surface reflection loss (SE_R_), internal absorption loss (SE_A_), and internal multiple reflection loss (SE_M_). SE_R_ is related to the impedance mismatch between material and free space, SE_A_ is determined by the material's inherent magnetic and dielectric loss capability to electromagnetic waves, and SE_M_ is the scattering effect of defects or non‐homogeneous interfaces within the material. Generally, the total electromagnetic shielding effectiveness (SE_T_) is as shown in Equation (5):

(5) $$SE_{T}=SE_{A}+SE_{R}+SE_{M}$$

when SE_T_ ≥ 15 dB, SE_M_ can be negligible. In a two‐port vector network analyzer, the scattering parameter S_ij_ represents the power transmitted from port i to port j. According to the S_ij_ can be calculated the reflection coefficient (R), transmission coefficient (T), and absorption coefficient (A). The calculation formulas are as follows:

(6) $$R={\left|S_{11}\right|}^{2}={\left|S_{22}\right|}^{2}\;$$

(7) $$T={\left|S_{21}\right|}^{2}={\left|S_{12}\right|}^{2}\;$$

(8) A=1−R−T

SE_R_, SE_A_, and SE_T_ can be deduced from the equation as follows:

(9) $$SE_{R}=10\;\log\left(\frac{1}{1-R}\right)=10\;\log\left(\frac{1}{1-{\left|S_{11}\right|}^{2}}\right)$$

(10) $$SE_{A}=10\;\log\;\left(\frac{1-R}{T}\right)=10\;\log\left(\frac{1-{\left|S_{11}\right|}^{2}}{{\left|S_{21}\right|}^{2}}\right)$$

(11) $$SE_{T}=10\;\log\;\left(\frac{1}{T}\right)=10\;\log\left(\frac{1}{{\left|S_{21}\right|}^{2}}\right)$$

## Conflict of Interest

The authors declare no conflict of interest.

## Supporting information



Supporting Information

## Data Availability

The data that support the findings of this study are available in the supplementary material of this article.
