# Supplementary material for: Breaking the Trade‐Off Between Electrical Conductivity and Mechanical Strength in Bulk Graphite Using Metal–Organic Framework‐Derived Precursors
Source: Adv Sci (Weinh). 2025 Jan 9;12(9):2416210. doi: 10.1002/advs.202416210 (PMC11884559; doi:10.1002/advs.202416210)
Supplement: Supplementary file 1 — Supporting Information [file ADVS-12-2416210-s001.docx]

Supporting Information

Breaking the Trade-Off between Electrical Conductivity and Mechanical Strength in Bulk Graphite Using Metal-Organic Framework-derived Precursors

Yuqing Zhang, Junzhuo Wang, Yinghan Zhang, Qi Zheng*, Lianjun Wang*, and Wan Jiang*


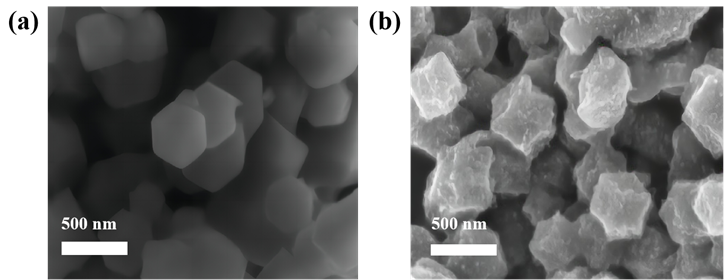


**Figure S1.** SEM images of ZIF-67(a)、C/Co(b).


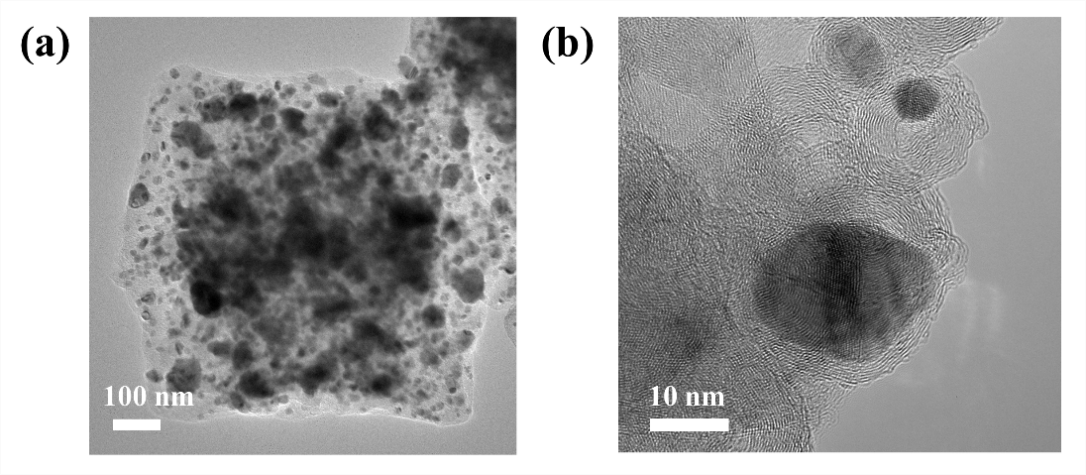


**Figure S2.** TEM (a) and HRTEM (b) images of C/Co.


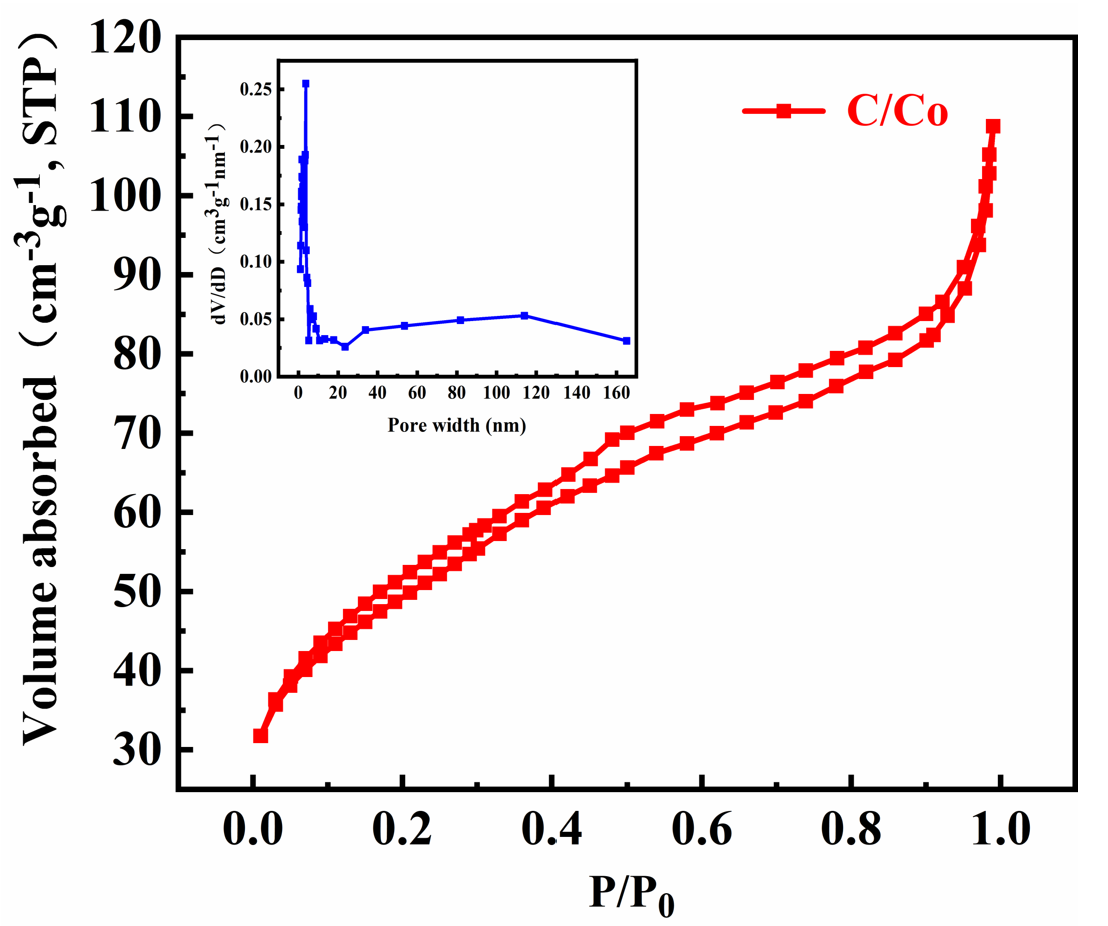


**Figure S3.** N_2_ adsorption-desorption isotherm curve and pore size distribution of C/Co.


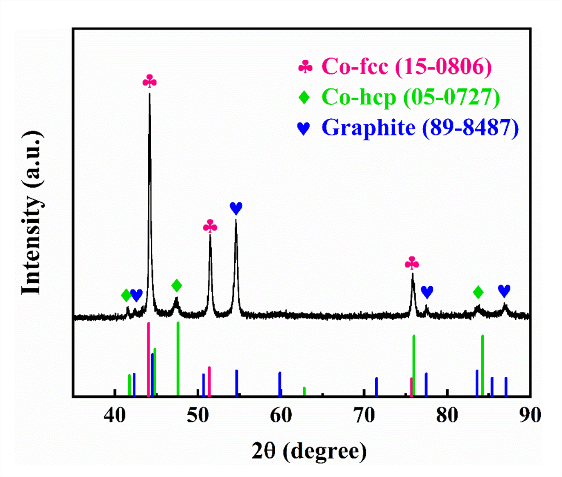


**Figure S4.** XRD pattern of C/CoB-1800.


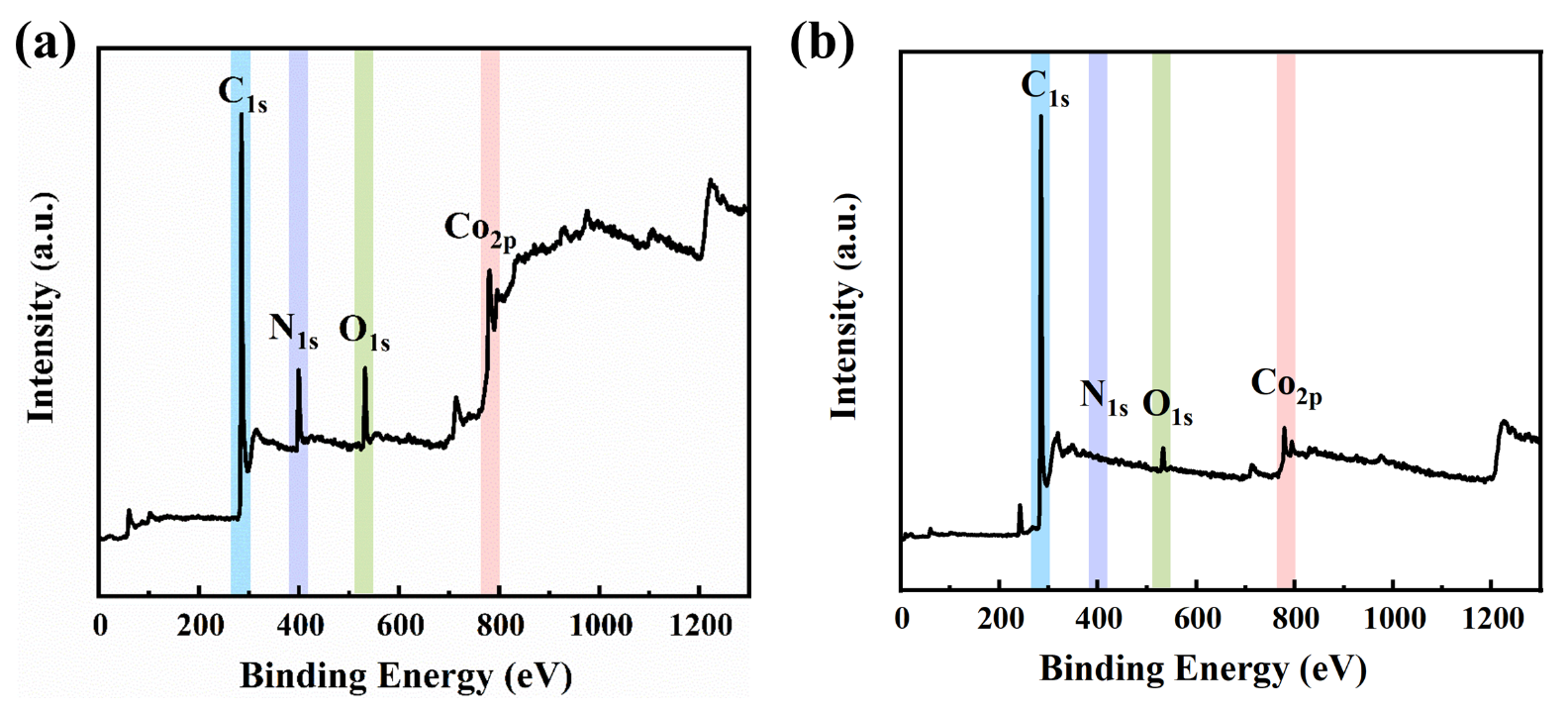


**Figure S5.** XPS survey spectra of C/Co (a) and C/CoB-1800 (b).


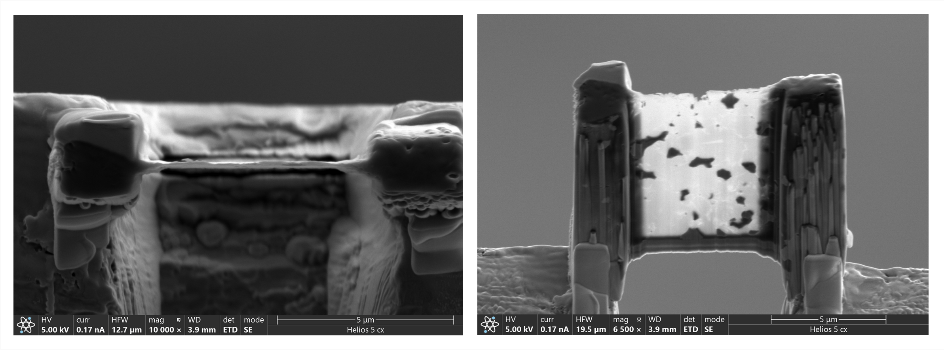


**Figure S6.** FIB-SEM images of C/CoB-1800.


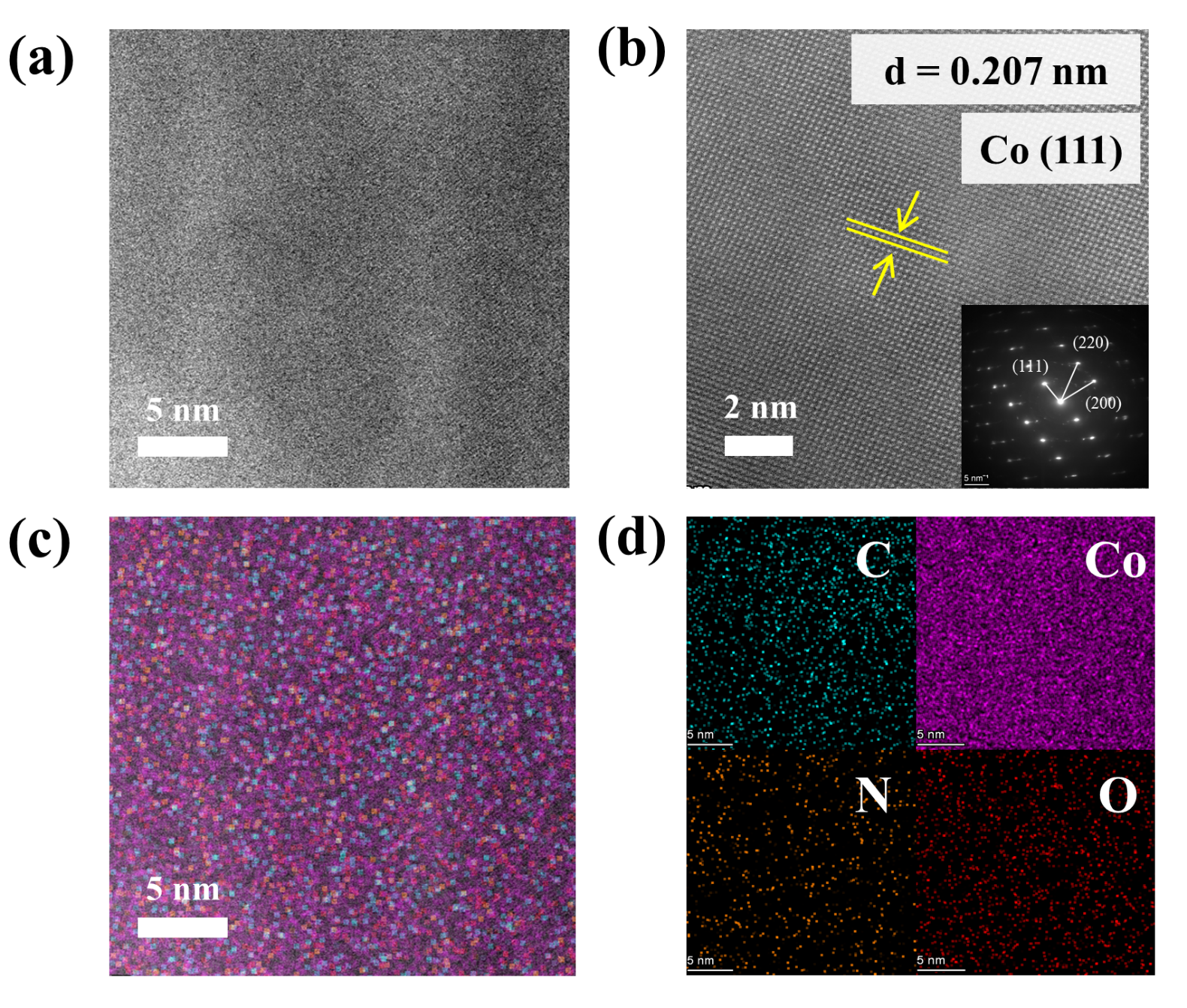


**Figure S7.** (a) HAADF-STEM image of Co nanoparticle regions in the C/CoB-1800. b) Detailed magnified HAADF-STEM with the selected area electron diffraction (SAED). c-d) EELS mapping with the distribution of Co, N, C and O.


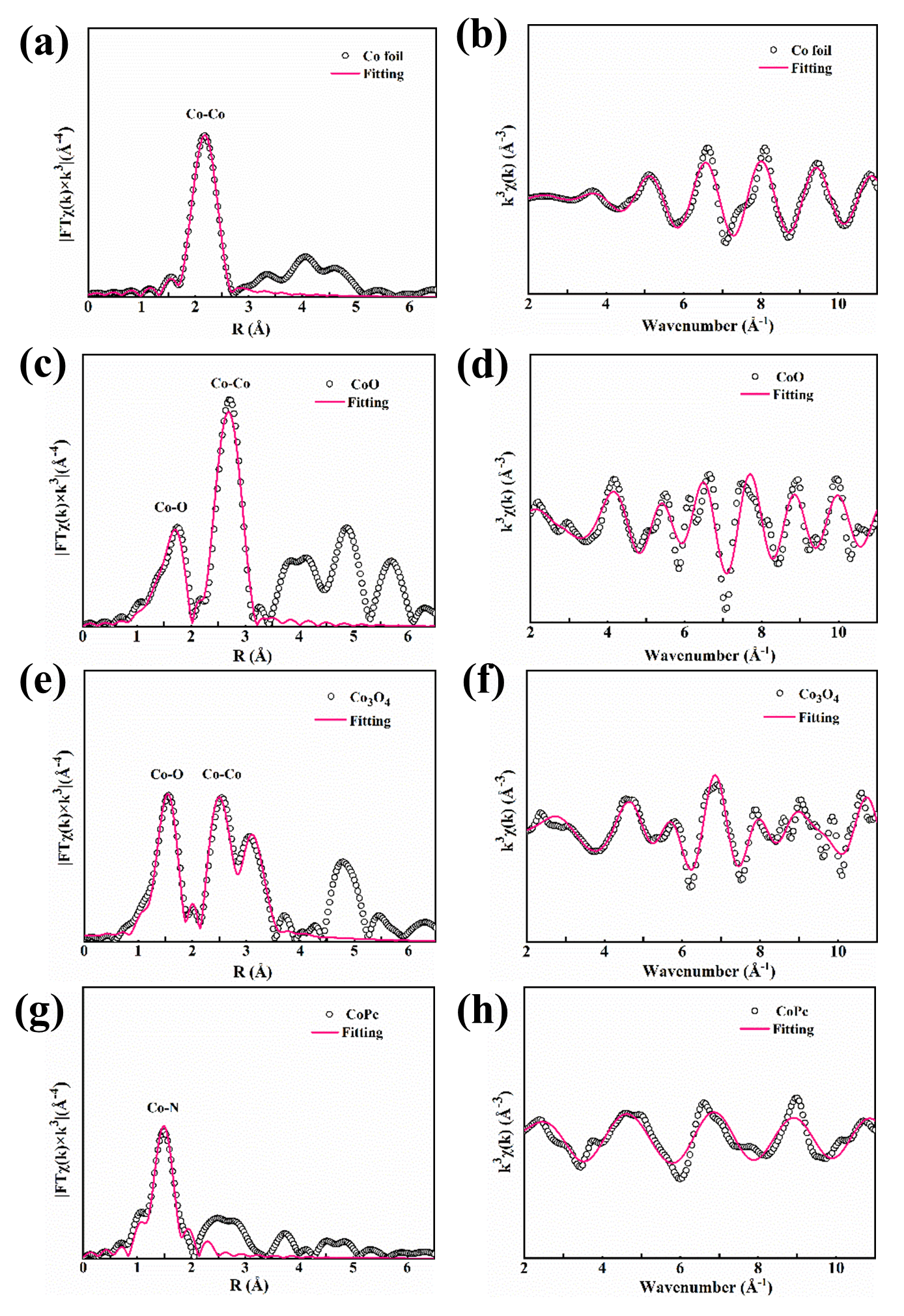


**Figure S8.** (a, c, e, g) The EXAFS fitting curves of Co foil, CoO, Co_3_O_4_, and CoPc in *K* space. (b, d, f, h) The EXAFS fitting curves of Co foil, CoO, Co_3_O_4_, and CoPc in *R* space.





**Figure S9.** Wavelet transform of Co_3_O_4_.





**Figure S10.** Young’s modulus-sintering temperature curves of C/CoB.





**Figure S11.** Stress-strain curves of C/CoB-1400, C/CoB-1600 and C/CoB-1800.


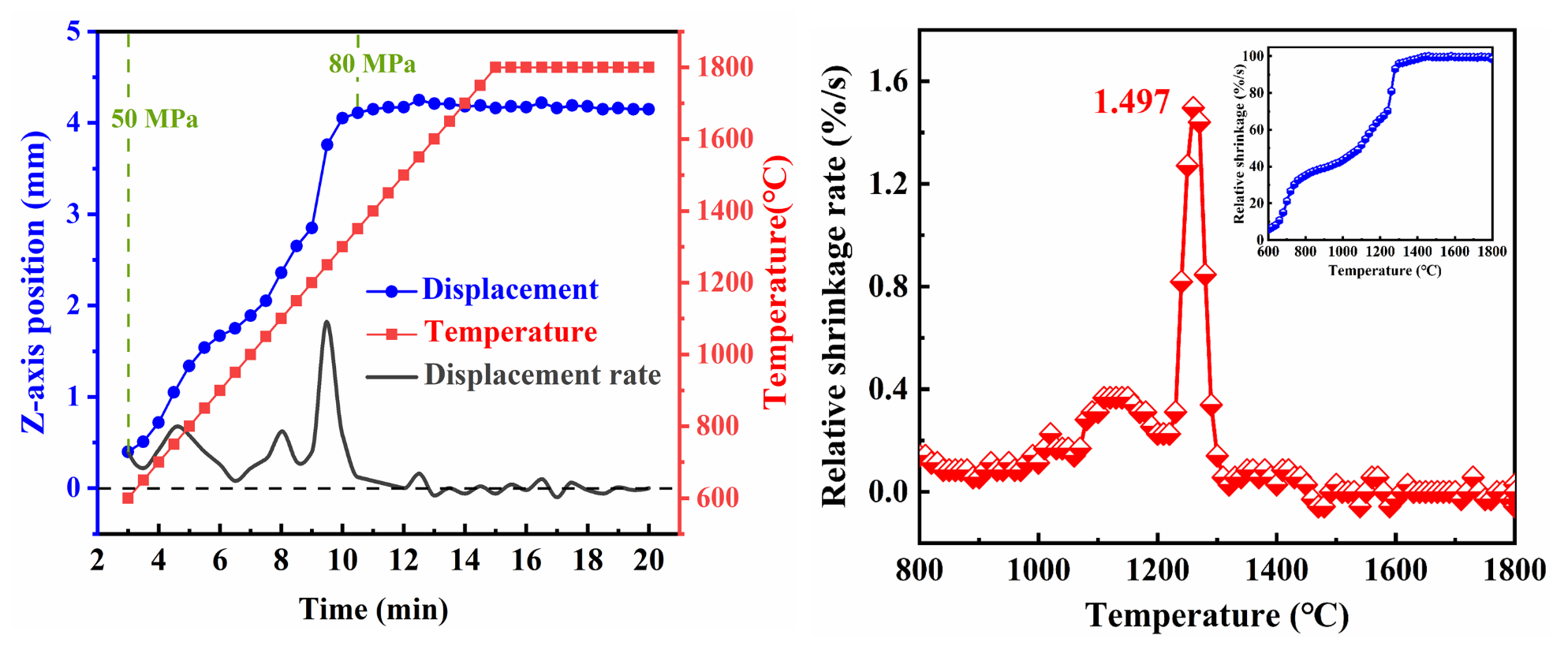


**Figure S12.** Sintering shrinkage curves of C/CoB-1800.


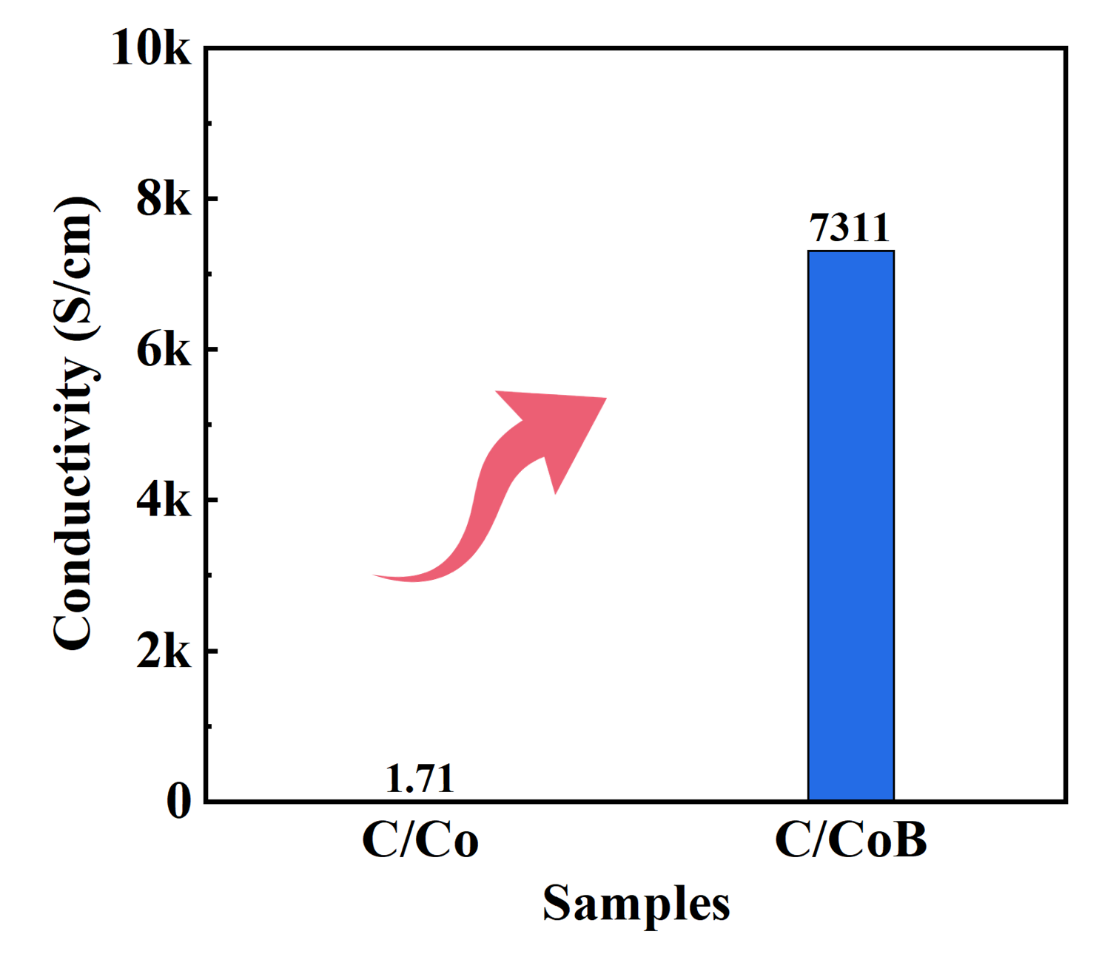


**Figure S13.** The electrical conductivity of C/Co and C/CoB-1800.


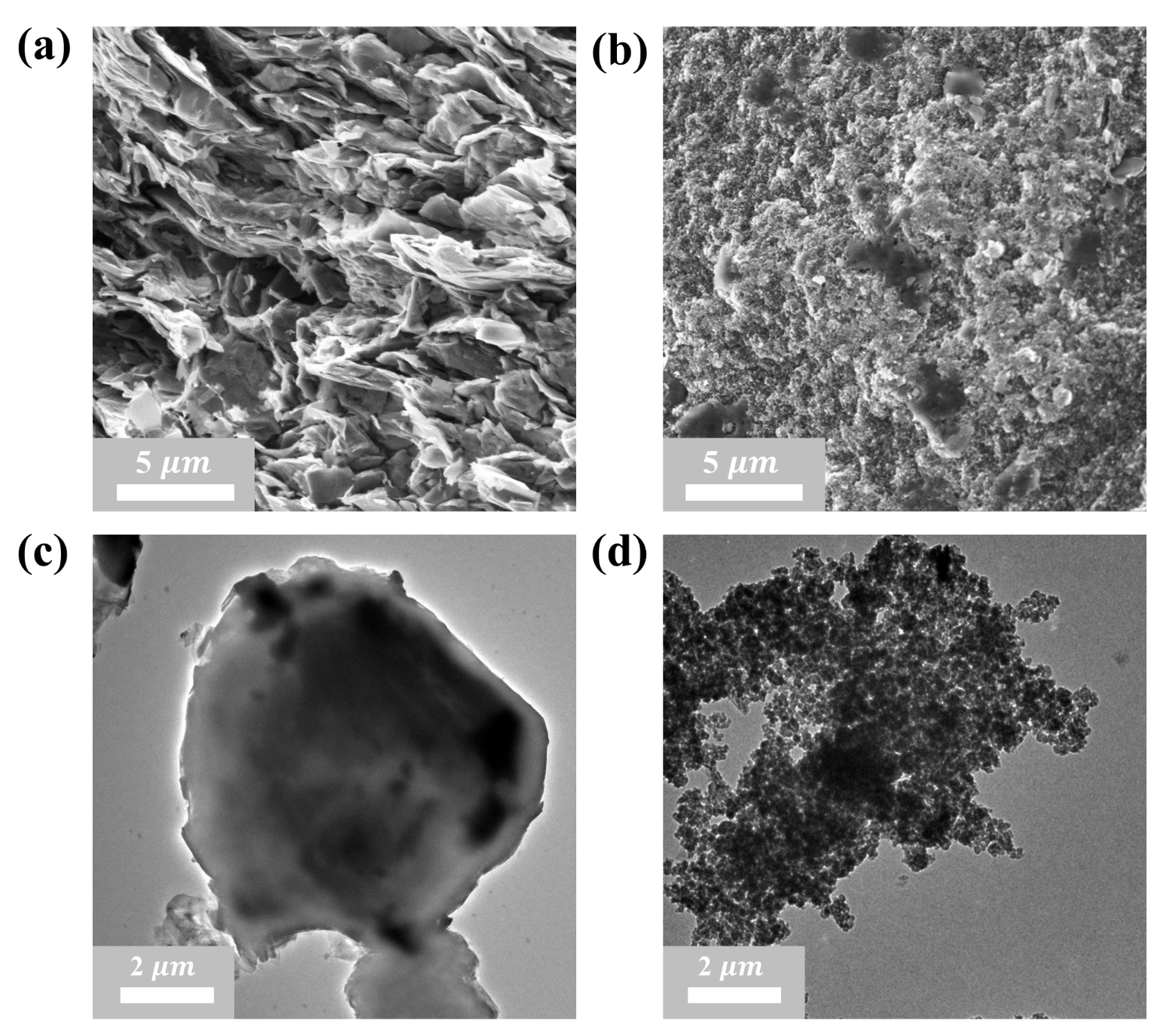


**Figure S14.** The cross-section SEM images of (a) GM-1 and (b) GM-2; TEM images of (c) GM-1 and (d) GM-2.


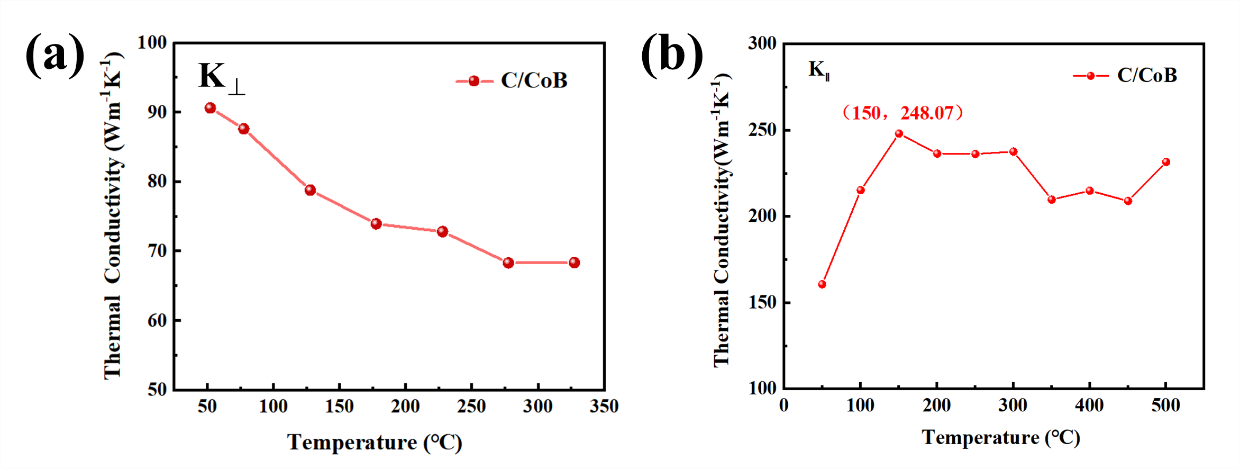


**Figure S15.** Temperature-dependent thermal conductivity curves of C/CoB-1800.


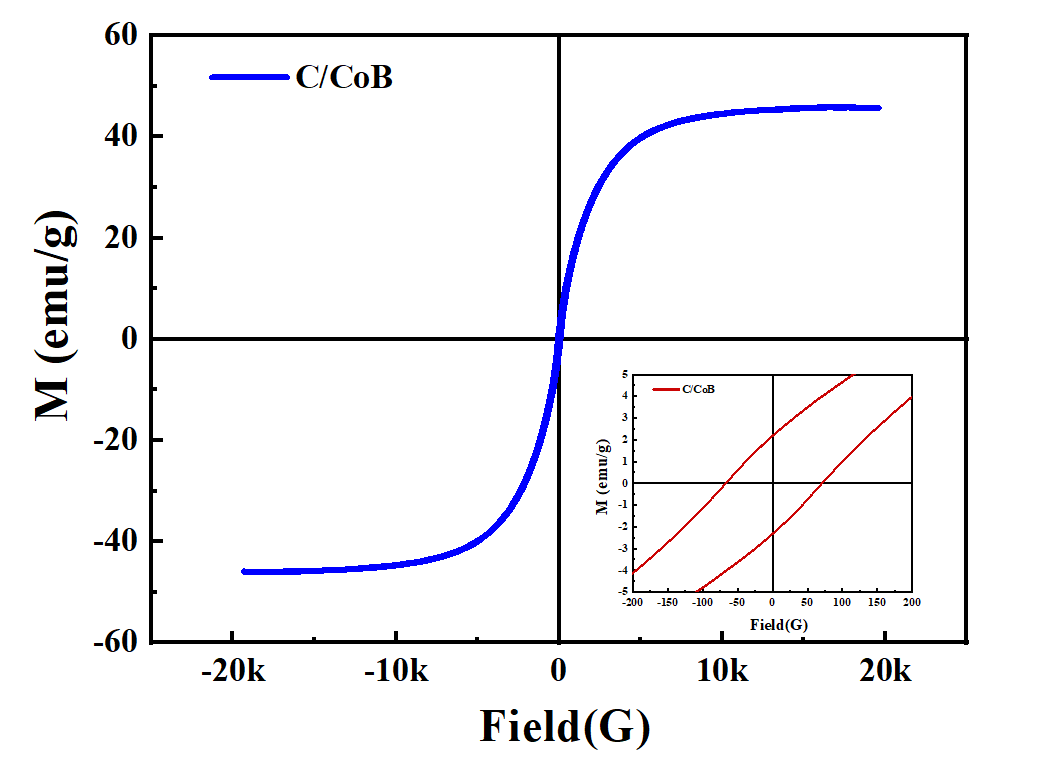


**Figure S16.** Magnetic hysteresis loops of C/CoB-1800.


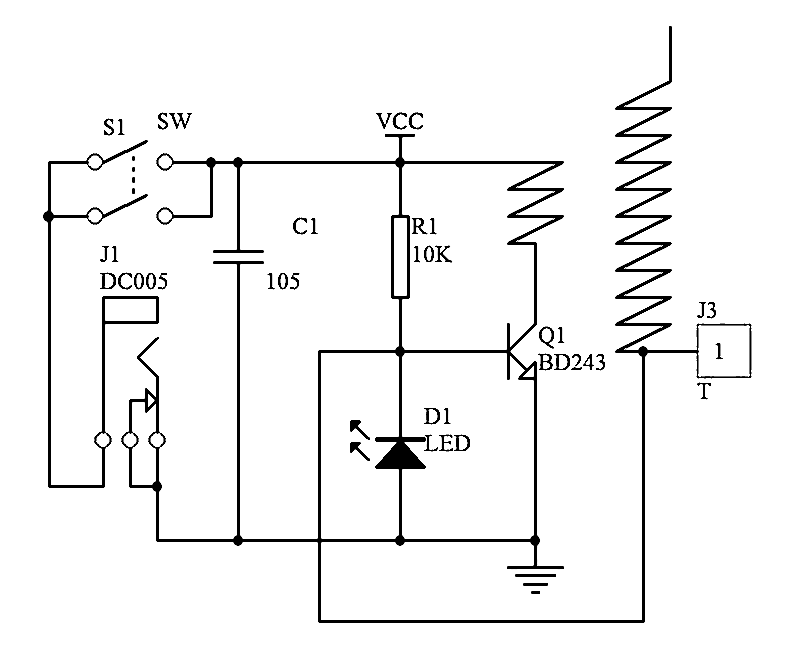


**Figure S17.** Tesla coil circuit schematic.

**Table S1.** EXAFS fitting parameters at the Co K-edge for various samples.

| Sample | Shell | | *CN^a^* | *R*(Å)*^b^* | *σ*^2^(Å^2^)*^c^* | Δ*E*_0_(eV)*^d^* | | *R* factor |
| --- | --- | --- | --- | --- | --- | --- | --- | --- |
| Co foil | | Co-Co | 12* | 2.49±0.01 | 0.0062 | | 7.30 | 0.0010 |
| CoO | | Co-O | 6.70±1.40 | 2.10±0.01 | 0.0086 | | 9.10 | 0.0198 |
|  |  | Co-Co | 15.40±2.20 | 3.00±0.01 | 0.0084 | |  |  |
| Co_3_O_4_ | | Co-O | 4.80±0.60 | 1.90±0.01 | 0.0022 | | 11.70 | 0.0181 |
|  |  | Co-Co | 5.70±1.30 | 2.86±0.01 | 0.0051 | |  |  |
|  |  | Co-Co | 7.10±2.00 | 3.37±0.01 | 0.0052 | |  |  |
| CoPc | | Co-N | 4.20±0.80 | 1.90±0.01 | 0.0018 | | 4.80 | 0.0150 |
| **Co sample** | | **Co-N** | **0.10±0.10** | **1.90±0.01** | **0.0053** | | **6.70** | **0.0159** |
|  |  | **Co-Co** | **3.10±0.10** | **2.48±0.01** | **0.0064** | |  |  |

*^a^CN*, coordination number; *^b^R*, distance between absorber and backscatter atoms; *^c^σ*^2^, Debye-Waller factor to account for both thermal and structural disorders; *^d^ΔE*_0_, inner potential correction; *R* factor indicates the goodness of the fit. S_0_^2^ was fixed to 0.715, according to the experimental EXAFS fit of Co foil by fixing CN as the known crystallographic value. A reasonable range of EXAFS fitting parameters: 0.600 < *Ѕ*_0_^2^ < 1.000; *CN >* 0; *σ*^2^ > 0 Å^2^; |Δ*E*_0_| < 15 eV; *R* factor < 0.02.

**Table S2.** The element content analysis of C/Co, C/CoB-1400, C/CoB-1600 and C/CoB-1800 using ICP-OES.

| Sample | C (%) | Co (%) | N (%) |
| --- | --- | --- | --- |
| C/Co | 70.11 | 19.77 | ≤0.05 |
| C/CoB-1400 | 55.16 | 28.01 | ≤0.05 |
| C/CoB-1600 | 64.30 | 27.28 | ≤0.05 |
| C/CoB-1800 | 66.94 | 23.73 | ≤0.05 |

**Table S3.** Electrical conductivity of MOF-derived bulk prepared at different sintering temperatures.

| Bulks | Electrical Conductivity (S cm^-1^) | Electrical Conductivity (S cm^-1^) |
| --- | --- | --- |
| C/CoB-1400 | ∥ 2810 | ⊥ 2535 |
| C/CoB-1600 | ∥ 3327 | ⊥ 2565 |
| C/CoB-1800 | ∥ 7731 | ⊥ 5541 |

**Table S4.** Comparative analysis of mechanical, electrical, and thermal properties in C/CoB-1800 and commercial HPBGs.

| Commercial HPBGs | Particle size  (μm) | Coefficient of thermal expansion  ×10^-6^ (K^-1^) | Compressive strength  (MPa) | Flexural strength  (MPa) | Conductivity (S cm^-1^) | Apparent density  (g cm^-3^) | Thermal conductivity  (W m^-1^K^-1^) | Ref. |
| --- | --- | --- | --- | --- | --- | --- | --- | --- |
| GF | ＜3 | 8.10 | 130 | 92 | 680 | 1.74 | 85 | [1] |
| XL | / | 8.10 | 162 | 95 | 568.10 | / | 77 | [2] |
| AFX-5QC | 5 | 8.70 | 210 | 117 | 4000 | 3.12 | 175 | [3] |
| ZEE-2 | 1 | 8.40 | 193 | 103 | 32.78 | 1.77 | 44 | [4] |
| HLM | / | ∥3, ⊥3.50 | ∥40, ⊥39 | ∥19, ⊥19 | ∥1282,  ⊥1053 | 1.72 | ∥160,  ⊥140 | [5] |
| WX-6 | 8 | 4.80 | 95 | 55 | 1250 | 1.90 | 140 | [6] |
| EDM-I3 | 18 | 4.20 | 80 | 37 | 833 | 1.80 | 110 | [7] |
| C/CoB-1800 | 0.30-0.50 | 4.50 | 151.56 | 101.17 | ∥7311,  ⊥5541 | 2.68 | ∥248,  ⊥91 | this work |

**Table S5.** The electrical conductivity, density and graphitization degree of GM-1, GM-2 and C/CoB-1800.

| Sample | Electrical conductivity  (S cm^-1^) | | | Density  (g cm^-3^) | Relative density | Graphitization degree |
| --- | --- | --- | --- | --- | --- | --- |
| GM-1 | ⊥951 | ∥1416 | 2.52 | | 89.40% | 96% |
| GM-2 | ⊥108 | ∥151 | 1.86 | | 66.00% | 54% |
| C/CoB-1800 | ⊥5541 | ∥7311 | 2.68 | | 92.52% | 98% |

**Table S6.** EMI SE values of C/CoB-1800 at X-, Ku- and K-band.

| Samples | X-band  8.2-12.4 GHz | | | Ku-band  12.4-18 GHz | | | K-band  18-26.5 GHz | | |
| --- | --- | --- | --- | --- | --- | --- | --- | --- | --- |
|  | SE_T_ | SE_A_ | SE_R_ | SE_T_ | SE_A_ | SE_R_ | SE_T_ | SE_A_ | SE_R_ |
| C/CoB-1800 | 68.26 | 51.35 | 16.91 | 53.52 | 35.47 | 18.05 | 32.45 | 17.61 | 14.84 |

**Table S7.** Comparative analysis of EMI shielding and electrical conductivity in C/CoB-1800 and typical carbon-based materials

| Samples | Conductivity (S cm^-1^) | Thickness (mm) | EMI SE_T_  (dB) | Frequency  (GHz) | Ref. |
| --- | --- | --- | --- | --- | --- |
| Cellulose/Biochar | 1.31 | 2.50 | 67.20 | X-band | [8] |
| ZIF-8/Poplar-CNT | 130 | 1.91 | 56.95 | X-band | [9] |
| Expanded graphite/epoxy | 71.50 | 2.70 | 85.00 | X-band | [10] |
| Expanded graphite/polyethylene | 40 | 2.00 | 52.40 | X-band | [11] |
| CNFs/CNTs | 54.20 | 0.20 | 40.00 | X-band | [12] |
| SiC_nf_-C/C | 326 | 2.00 | 27.86 | X-band | [13] |
| POM/PLLA/MWCNT | 3.33 | 2.00 | 29.30 | X-band | [14] |
| Cu-Ni wood-based composites | 1930 | 0.40 | 57.40 | X-band | [15] |
| Conductive ceramic | 0.22 | 5.00 | 26.20 | X-band | [16] |
| C/SiC | 64.40 | 0.41 | 56.76 | X-band | [17] |
| C/CoB-1800 | 7311 | 0.74 | 68.26 | X-band | this work |

**Table S8.** EMI SE_T_ values of the typical carbon-based materials at Ku- and K-band.

| Samples | Thickness (mm) | EMI SE_T_ (dB) | | Ref. |
| --- | --- | --- | --- | --- |
|  |  | Ku-band | K-band |  |
| Expanded graphite/Silicone | 0.84 | 20.50 | / | [18] |
| Coal | 2.74 | 50~62.20 | / | [19] |
| Graphene-based Bulk | 3.00 | 48 | / | [20] |
| CNF/Epoxy | 0.50 | 35 | / | [21] |
| ZrO_2_/CNF/Epoxy | 2.00 | 22~26 | / | [22] |
| MXene/Carbon | 2.00 | 30 | 33 | [23] |
| rGO/SiC | 1.00 | 40 | 40 | [24] |
| C/CoB-1800 | 0.74 | 53.52 | 32.45 | this work |

**Table S9.** Comparative analysis of EMI shielding at X-band and compressive strength of C/CoB-1800 and typical carbon-based EMI shielding bulks.

| Samples | Electrical conductivity (S cm^-1^) | Compressive strength (MPa) | EMI SE_T_  (dB) | Ref. |
| --- | --- | --- | --- | --- |
| FeCl_3_/PPy/wood | 0.39 | 15.46 | 58.00 | [25] |
| Ni-CNTs/PLA | 7.58×10^-2^ | 5.42 | 25.20 | [26] |
| Graphene/ADCN | 1.20 | 143.00 | 47.00 | [27] |
| Carbonized wood | 4.60 | 40.22 | 58.55 | [28] |
| C/CoB-1800 | 7311 | 151.56 | 68.26 | this work |

**Table S10.** The joule heating properties of the carbon-based composite materials.

| Samples | Voltage | Maximum temperature (°C) | Ref. |
| --- | --- | --- | --- |
| Ni@NCNT | 1V | 38 | [29] |
| CNFs/Silicone | 1V | 49 | [30] |
| Graphene/Fe_3_O_4_ | 1V | 35 | [31] |
| Bamboo-based derived carbon | 1V | 50 | [32] |
| CNT sponge | 1V | 53 | [33] |
| Carbonized wood/Fe-MOF | 1V | 35 | [34] |
| MOF-74/CNF/MXene | 1V | 32.90 | [35] |
| Carbonized wood/ZIF-8 | 4V | 70 | [36] |
| Expanded graphite/Silicone | 5V | 54 | [18] |
| C/CoB-1800 | 1V | 80.20 | This work |

**References**

1. P. M. Entegris, Glass Forming Graphite Grades and performance parameters, https://www.entegris.com/shop/en/USD/Products/Specialty-Materials/Premium-Graphite/Glass-Forming-Graphite-Grades/c/glass forming graphite grades, accessed: **2024.**
2. P. M. Entegris, Typical Graphite Grade Matrial Properties, https://poco.entegris.com/content/dam/poco/resources/reference-materials/data-sheets/datasheet-glassmate-graphite-10196.pdf, accessed: **2024.**
3. P. M. Entegris, Industrial Graphite Grades and performance parameters, https://poco.entegris.com/en/home/products/premium-graphite/industrial-grades.html, accessed: **2024.**
4. P. M. Entegris, Semiconductor Graphite Grades and performance parameters, https://poco.entegris.com/en/home/products/premium-graphite/semiconductor-grades.html, accessed: **2024.**
5. SGL carbon, Molded Carbon and Graphite, https://www.sglcarbon.com/en/markets-solutions/material/sigrafine-die-molded-carbon-and-graphite/, accessed: **2024.**
6. Henan wuxing New Material Science and Technology Inc, High purity graphite

bulk, https://www.wxxc.com/product_detail/48.html, accessed: **2024.**

1. Chengdu carbon material, EMD graphite processing, http://www.cdcarbon.cn/product/22/, accessed: **2024.**
2. S. Li, Y. Du, H. Ye, J. Wu, Y. Wang, Y. Liang, M. Zhu, S. S. Lam, C. Liu, J. Li, C. Xia, *Adv. Funct. Mater.* **2024**, *34*, 2406282.
3. H. Ye, Y. Wu, X. Jin, J. Wu, L. Gan, J. Li, L. Cai, C. Liu, C. Xia, *Adv. Sci*. **2024**, *11*, 2400074.
4. D. Bao, Y. Gao, Y. Cui, F. Xu, X. Shen, H. Geng, X. Zhang, D. Lin, Y. Zhu, H. Wang, *Chem. Eng. J.* **2022**, *433*, 133519.
5. B. Wei, L. Zhang, S. Yang, *Chem. Eng. J.* **2021**, *404*, 126437.
6. X. Feng, X. Wang, M. Wang, S. Tao, Y. Chen, H. Qi, *J. Mater. Chem. A*. **2022**, *10*, 22271.
7. Z. Zhang, L. Cheng, T. Sheng, J. Yu, J. Tan, W. Yang, *ACS Appl. Nano Mater*. **2022**, *5*, 195.
8. J. Li, J.-L. Chen, X.-H. Tang, J.-H. Cai, J.-H. Liu, M. Wang, J*. Colloid Interface Sci*. **2020**, *565*, 536.
9. Y. Pan, M. Dai, Q. Guo, D. Yin, S. Hu, N. Hu, X. Zheng, J. Huang, *Chem. Eng. J.* **2023**, *471*, 144301.
10. D. Li, B. Tang, D. Cheng, J. Wu, W. Tang, Z. Zhao, J. Li, G. Cai, J. Wang, X. Wang, *Engineering* **2023**, *21*, 143.
11. Z. Zhang, L. Cheng, J. Tan, W. Yang, *Ceram. Int.* **2021**, *47*, 23942.
12. Y. Dong, X. Yuan, *Polym. Compos.* **2024**, *1–9*.
13. Y. Huang, S. Chen, R. Ma, Y. Cheng, L. Jin, G. Chen, *Adv. Compos. Hybrid Mater.* **2022**, *5*, 2193.
14. B. D. S. Deeraj, G. George, N. R. Dhineshbabu, S. Bose, K. Joseph, *Mater. Res. Bull.* **2021**, *144*, 111477.
15. L. Mohan, T. N. Kumar, S. Karakkad, S. T. Krishnan, *IEEE Trans. Nanotechnol.* **2021**, *20*, 627.
16. G. Parameswarreddy, A. Vinayakumar, V. Subramanian, R. Sarathi, *Polym. Compos.* **2022**, *43*, 8795.
17. Y. Lu, X. Zhao, Y. Lin, P. Li, Y. Tao, Z. Wang, J. Ma, H. Xu, Y. Liu, *Carbon.* **2023**, *206*, 375.
18. X. He, L. Feng, Z. Zhang, X. Hou, X. Ye, Q. Song, Y. Yang, G. Suo, L. Zhang, Q. G. Fu, H. Li, *ACS Nano.* **2021**, *15*, 2880.
19. W. Gan, C. Chen, M. Giroux, G. Zhong, M. M. Goyal, Y. Wang, W. Ping, J. Song, S. Xu, S. He, M. Jiao, C. Wang, L. Hu, *Chem. Mater.* **2020**, *32*, 5280.
20. T. Kuang, J. Ju, F. Chen, X. Liu, S. Zhang, T. Liu, X. Peng, *Compos Sci Technol.* **2022**, *230*, 109736.
21. H. Xiao, T. Zhou, J. Lv, X. He, M. Chen, K. Zeng, J. Hu, G. Yang, *J. Electron. Mater.* **2022**, *51*, 5120.
22. Z. Dai, Y. Wei, C. Hu, Z. Wang, A. Wei, W. Zhang, X. Lin, *Compos. Commun.* **2023**, *38*, 101501.
23. X. Hao, D. Li, X. Peng, W. Lan, C. Liu, *Chem. Eng. J.* **2024**, *479*, 147681.
24. Z. Li, Z. Lin, M. Han, Y. Mu, P. Yu, Y. Zhang, J. Yu, *Chem. Eng. J.* **2021**, *420*, 129826.
25. Z. Yu, W. Yu, Y. Jiang, Z. Wang, W. Zhao, X. Liu, *ACS Appl. Nano Mater.* **2022**, *5*, 13158.
26. C. Wang, X. Lin, J. Xu, A. Wei, Z. Wang, W. Zhang, C. Hu, *Carbon.* **2024**, *233*, 119872.
27. S. Li, C. Tang, Y. Song, S. Zhang, Z. H. Hang, X. Zhang, Y. Li, Z. Yang, *ACS Appl. Mater. Interfaces.* **2024**, *16*, 11821.
28. Y. Rong, X. Zhou, S. Jiang, W. Zhu, H. Wang, B. Dang, Q. Sun, X. Ma, *Chem. Eng. J.* **2025**, *503*, 158556.
29. Z. Guo, Y. Zhao, P. Luo, J. Wang, L. Pei, H. Yu, P. Song, F. Ren, P. Ren, *Chem. Eng. J.* **2024**, *497*, 155707.
30. X. Ma, J. Pan, H. Guo, J. Wang, C. Zhang, J. Han, Z. Lou, C. Ma, S. Jiang, K. Zhang, *Adv. Funct. Mater.* **2023**, *33*, 2213431.
